# Organoid Intelligent Morphomics: Decoding the organoid morphome through artificial intelligence from phenotypic quantification to mechanistic insight

**DOI:** 10.1016/j.bioactmat.2026.08.043

**Published:** 2026-09-03

**Authors:** Shangyan Li, Jiqing Huang, Yongyong Yan, Richard T. Jaspers, Janak Lal Pathak, Yin Xiao, Qing Zhang

**Affiliations:** a Department of Basic Oral Medicine, School and Hospital of Stomatology, Guangdong Engineering Research Center of Oral Restoration and Reconstruction, Guangzhou Key Laboratory of Basic and Applied Research of Oral Regenerative Medicine, Guangzhou Medical University, Guangzhou, 510182, China; b School of Medicine and Dentistry & Institute for Biomedicine and Glycomics, Griffith University, Gold Coast, QLD, 4222, Australia; c The Australia-China Centre for Tissue Engineering and Regenerative Medicine (ACCTERM), Brisbane, QLD, 4000, Australia; d Laboratory for Myology, Department of Human Movement Sciences, Faculty of Behavioural and Movement Sciences, Vrije Universiteit Amsterdam, Amsterdam Movement Sciences, Amsterdam, 1081 BT, the Netherlands

**Keywords:** Organoid, Morphomics, Deep learning, Image analysis, Phenotypic analysis

## Abstract

Organoids, as three-dimensional bioactive microtissues capable of recapitulating native organ architecture and function, have become valuable models in biomedical research. However, their broad adoption is constrained by subjective morphological assessment and endpoint assays that compromise standardization and scalability. This review introduces Organoid Intelligent Morphomics (OIM), an integrative analytical framework that synergizes imaging technologies with artificial intelligence to establish a “morphology–function–mechanism” mapping. OIM enables a paradigm shift from qualitative to quantitative analysis, from static to dynamic monitoring, and from superficial observation to deep molecular inference. The applications of OIM are critically examined across three interconnected domains: (1) quality control, encompassing real-time assessment through non-invasive viability quantification and morphological evaluation, as well as predictive quality control for early differentiation outcome forecasting; (2) disease deconstruction, enabling quantitative, multiscale phenotyping of disease morphology and establishing morphology–mechanism association analyses; (3) drug development, facilitating high-throughput efficacy screening, toxicity assessment, and personalized therapeutic guidance. Furthermore, we critically analyze the current data, algorithmic, and translational challenges facing OIM, propose corresponding solutions, and provide a roadmap toward artificial intelligence virtual organoids. Ultimately, OIM holds promise for establishing the critical technological infrastructure for the scalable manufacturing and clinical translation of organoid-based therapeutics.

## Highlights

• Organoid Intelligent Morphomics (OIM) is an integrative analytical framework.
• Imaging technologies and AI are the technical foundations of OIM.
• OIM transforms organoid morphology into a quantitative, predictive science.
• Key applications are quality control, disease deconstruction, drug development.
• OIM provides a foundation for future predictive virtual organoids.

## Graphical abstract

**Figure:**
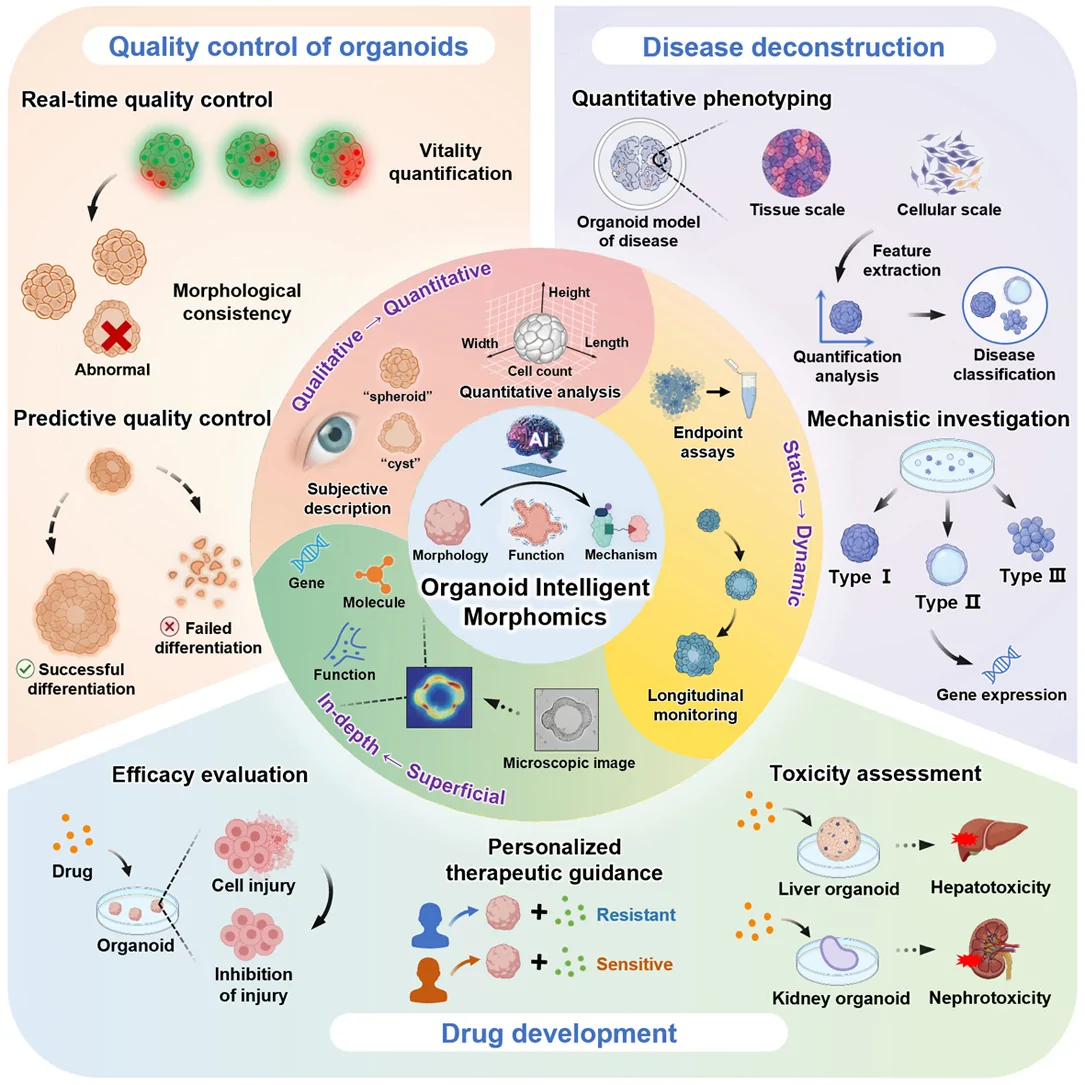

## 1 Introduction

Organoids, three-dimensional (3D) microtissues derived from stem cell self-organization, can faithfully recapitulate the structural and functional complexity of native organs *in vitro* and have become valuable models in biomedical research [1,2]. Nevertheless, a fundamental challenge persists: how can we fully decode the biological information embedded within organoids? Traditionally, organoid analysis has predominantly relied on two complementary yet limiting methodologies. First, endpoint molecular assays deliver high-resolution molecular profiles but require sample destruction, precluding longitudinal observation [3]. Second, manual visual analysis allows for non-destructive monitoring but suffers from subjective interpretation, low throughput, and qualitative categorization [4]. While emerging fields such as radiomics and computational pathology have provided robust quantitative paradigms, they are primarily optimized for macroscopic medical imaging or static histological sections [5]. Consequently, they are fundamentally limited in their capacity to capture the entire culture lifespan and dynamic evolution of living organoids.

Morphology is a cornerstone of phenomics, playing a pivotal role in reflecting the fundamental life activities of biological systems [6]. The totality of morphological features within a biological system constitutes the morphome, and the systematic study of these features is defined as morphomics [7]. Organoid morphomics not only facilitates the linkage of phenotypes to underlying biological mechanisms but also offers potential to support prediction of future physiological state transitions through morphological analysis [8]. However, the practical implementation of organoid morphomics is hindered by immense analytical challenges. While the advent of high-content imaging (HCI) and high-content screening (HCS) platforms featuring automated image acquisition and predefined feature extraction has dramatically enhanced experimental throughput and data standardization [9,10], it has paradoxically created a profound data bottleneck. The generated datasets exhibit a volume, dimensionality, and temporal dynamism that substantially exceed the processing capabilities of conventional manual approaches [11,12].

To bridge this critical gap, artificial intelligence (AI) technologies, particularly the breakthroughs in deep learning (DL) for computer vision, provide crucial technical means for addressing this analytical complexity [[13], [14], [15]]. Unlike traditional image analysis methods that depend on handcrafted features, AI-based image analysis can automatically learn hierarchical, non-linear feature representations directly from raw imaging data [16,17]. This capability enables systematic, quantitative, large-scale morphological characterization of organoids and facilitates deeper biological interpretation.

In this context, we formally propose Organoid Intelligent Morphomics (OIM) as an integrative analytical framework (Table S1). Rather than representing a single imaging modality or AI algorithm, OIM integrates standardized morphological data acquisition, AI-driven multidimensional morphome construction, morphology–biology association and predictive modelling, and biological validation to systematically decode the organoid morphome across both spatial and temporal dimensions. This distinguishes OIM from conventional image analysis approaches that primarily focus on morphological quantification without explicitly integrating functional prediction and mechanistic validation. Its ultimate objective is to establish a hierarchical “morphology–function–mechanism” mapping, progressing from morphological quantification to functional correlation or prediction and, where supported by experimental evidence, mechanistic validation. By systematically linking morphological alterations with functional states and underlying biological drivers, OIM extends conventional image analysis beyond feature measurement toward biologically interpretable and predictive organoid analysis. Ultimately, this framework catalyzes a fundamental paradigm shift in organoid research, driving the transition from qualitative, static, and descriptive observations to quantitative, dynamic, and mechanistically insightful investigations.

**Fig. 1:**
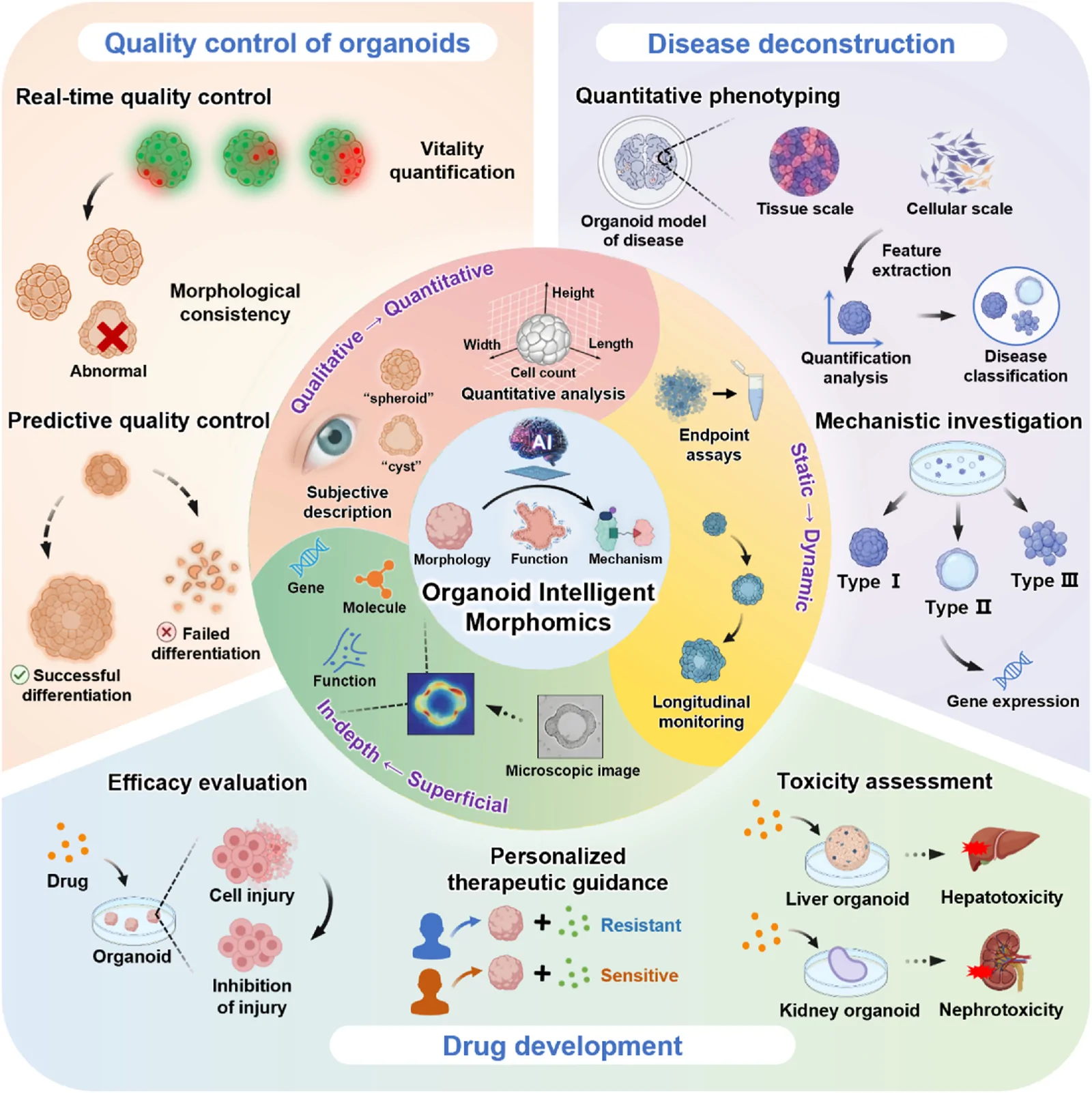
Schematic overview of OIM. OIM aims to establish a “morphology–function–mechanism” mapping, thereby driving organoid research from qualitative to quantitative, from static to dynamic, and from superficial to in-depth analyses. It has demonstrated applications in organoid quality control, disease deconstruction, and drug development.

This review establishes a comprehensive theoretical framework and methodological roadmap for OIM, systematically presenting its technical foundations, core methodologies, current applications (Fig. 1), while critically discussing existing challenges and future perspectives. By promoting the paradigm shift from empirical to data-driven approaches in organoid research, this work bridges the critical gap between morphological analysis and mechanistic understanding.

## 2 Technical foundations of OIM: data acquisition and intelligent analysis

These two technologies synergistically drive the advancement of OIM: imaging technology captures the complex morphology of organoids, establishing the data foundation for subsequent analysis, whereas AI algorithms process image data to uncover underlying biological patterns. Their synergistic progression is evident: imaging advances generate higher-dimensional and higher-resolution data, propelling the evolution of analytical tools; conversely, AI breakthroughs extract increasingly rich biological insights from complex morphological features. This chapter provides an in-depth examination of the evolutionary trajectories and pivotal breakthroughs of these two foundational technologies.

### 2.1 Imaging technologies: establishing the data foundation for intelligent analysis

**Fig. 2:**
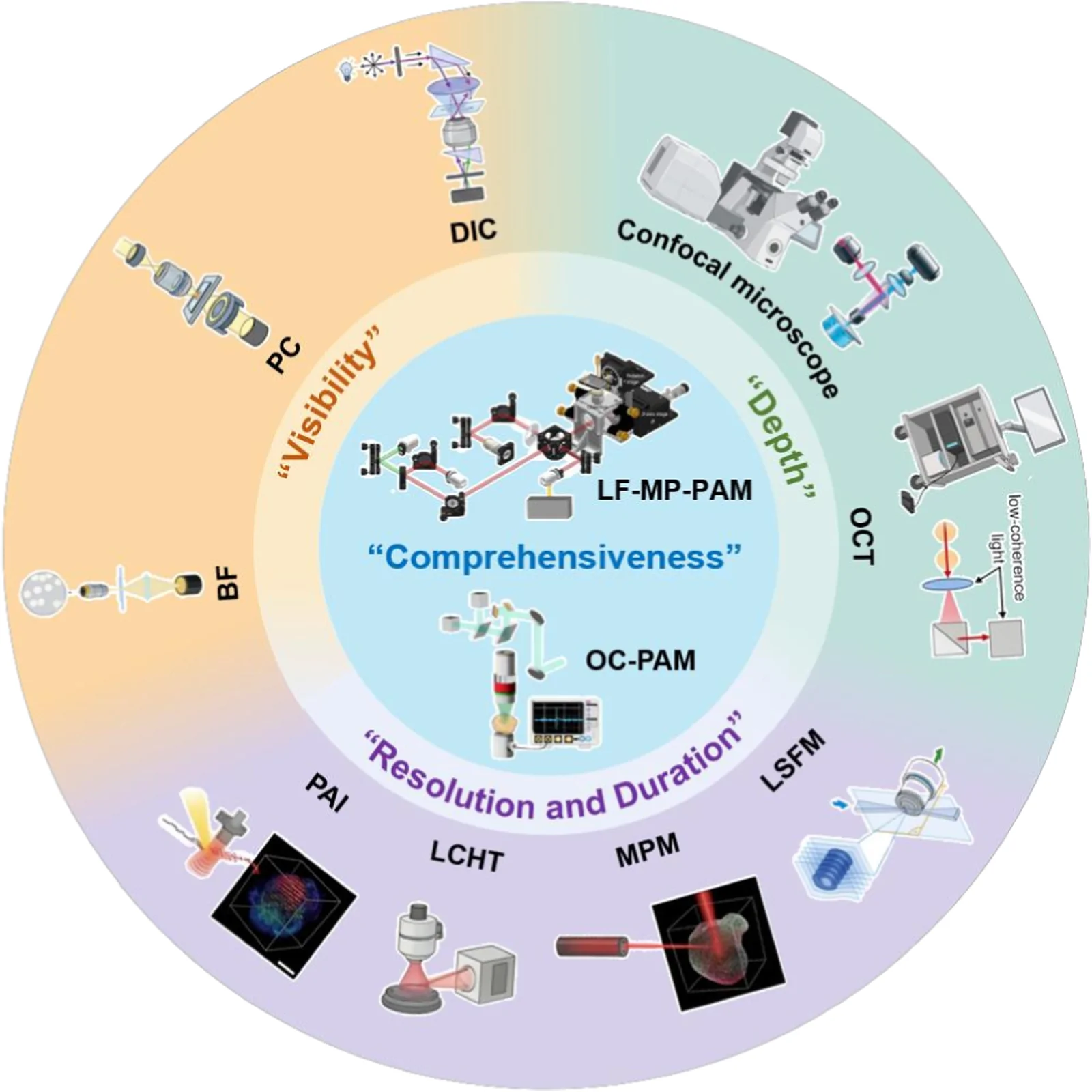
Integrated multimodal imaging technologies for organoid analysis. The framework comprises four modules: **Module 1 (Visibility)**: Basic imaging techniques focused on rendering organoid morphology visible, such as BF, PC, and DIC [18,19]; **Module 2 (Depth)**: 3D imaging techniques including CLSM [20,21] and OCT [22], which address the challenge of imaging depth; **Module 3 (Resolution and Duration)**: Non-destructive volumetric imaging with high resolution and long-term capability achieved through PAI [23,24], LCHT [25], MPM [26], and LSFM [[27], [28], [29]]; **Module 4 (Comprehensiveness)**: Multimodal imaging platforms, such as LF-MP-PAM [30] and OC-PAM [31], integrate complementary imaging modalities for more comprehensive observation of organoids. **Abbreviations:** BF, brightfield microscopy; PC, phase contrast microscopy; DIC, differential interference contrast microscopy; CLSM, confocal laser scanning microscopy; OCT, optical coherence tomography; PAI, photoacoustic imaging; LCHT, low-coherence holographic tomography; MPM, multiphoton microscopy; LSFM, light-sheet fluorescence microscopy; LF-MP-PAM, label-free multiphoton photoacoustic microscopy; OC-PAM, optical coherence photoacoustic microscopy.

The intricate 3D architecture of organoids presents significant challenges for imaging technologies. Consequently, the quality of image data produced by these technological innovations directly determines the reliability of downstream intelligent analysis. The mutual evolution and organic integration of various imaging modalities have ultimately driven the formation of multimodal imaging systems. These systems can be categorized into four modules: “visibility”, “depth”, “resolution and duration” and “comprehensiveness” (Fig. 2).

**Module 1: 2D widefield imaging for rendering organoid morphology visible.** Early organoid research has primarily employed 2D widefield microscopy techniques such as brightfield (BF), phase contrast (PC), and differential interference contrast (DIC) microscopy [18,19]. These methods are operationally simple, non-destructive, and highly amenable to rapid image acquisition, generating relatively small datasets with modest computational requirements. Such advantages have made 2D imaging particularly suitable for large-scale and routine assessment of organoid biology, including population-level morphological consistency, gross growth and size dynamics [32,33]. However, these benefits are accompanied by an intrinsic loss of 3D spatial information. Because 2D imaging captures organoids primarily as planar projections, it provides limited access to their internal architecture and spatially resolved cellular features [34,35]. Moreover, out-of-focus signals in thick organoid specimens can obscure in-focus structures, reducing image contrast and compromising the accurate delineation of internal architectural features [34]. These intrinsic limitations have established the primary objective for subsequent technological advancement: high-fidelity 3D reconstruction.

**Module 2: 3D structural imaging for addressing the challenge of imaging depth.** Volumetric imaging is particularly important for addressing biological questions that depend on 3D spatial organization, including complex tissue cytoarchitecture and layer-specific organization [36], spatial heterogeneity of cell–cell interactions [37], 3D lumen architecture and luminal volume [38]. To obtain 3D structural information, technological pathways have branched into two complementary approaches. The first, represented by confocal laser scanning microscopy (CLSM), provides high molecular specificity through fluorescence labeling but is limited by phototoxicity and photobleaching, making it suboptimal for continuous, long-term live imaging [20,21]. The second, exemplified by OCT [22], enables label-free 3D reconstruction of organoid architecture but lacks resolution at the cellular level. Consequently, the field was propelled into a new phase: balancing high spatial resolution with minimized photodamage to sustain continuous 3D tracking over extended timeframes.

**Module 3: Non-destructive volumetric imaging to balance resolution and duration.** To reconcile the demands for both high resolution and long-term observation, several non-destructive, high-penetration imaging modalities have emerged. In terms of enhancing imaging depth, label-free photoacoustic imaging (PAI) has overcome the trade-off between depth of field and resolution in traditional photoacoustic imaging, enabling high-resolution 3D observation of organoids [23,24]. Similarly, low-coherence holographic tomography (LCHT) achieves subcellular resolution through thick samples, allowing quantitative monitoring of organoid growth and drug-induced morphological changes [25]. To address phototoxicity concerns, multiphoton microscopy (MPM) [26] and light-sheet fluorescence microscopy (LSFM) [[27], [28], [29]] significantly reduce photodamage, thereby enabling continuous 3D imaging of thick organoids over days to weeks. Nevertheless, no single modality can simultaneously provide optimal imaging depth, spatial resolution, longitudinal compatibility, and molecular or metabolic specificity. This limitation has motivated the development of multimodal imaging strategies that combine complementary capabilities.

**Module 4: Multimodal imaging for comprehensive observation.** Advanced multimodal imaging systems, such as label-free multiphoton photoacoustic microscopy (LF-MP-PAM) [30] and optical coherence photoacoustic microscopy (OC-PAM) [31], synergistically integrate multiple imaging modalities, including optical, photoacoustic, and coherent interference techniques, within a unified platform. These integrated systems enable simultaneous acquisition of multidimensional information encompassing organoid structure, molecular functionality, and metabolic dynamics, representing a significant technological advancement toward the goal of ideal imaging [30,31]: label-free, non-destructive, volumetric imaging with subcellular resolution, longitudinal tracking, and high-throughput capability.

Collectively, the evolution of imaging technologies has followed a logical trajectory from 2D to 3D, from potentially invasive to non-destructive imaging, and from single-modality to multimodal integration, progressively increasing the biological information content of OIM data. Importantly, richer biological information is accompanied by a practical trade-off with imaging throughput, as higher-dimensional and multimodal acquisition generally requires longer acquisition times, larger datasets, and greater experimental and computational resources. Therefore, high-throughput 2D imaging remains advantageous for population-scale screening and routine quality control, whereas volumetric imaging is required when biological questions specifically depend on quantitative 3D information, while multimodal approaches provide additional molecular, functional, or metabolic information when needed. For example, planar measurements are generally sufficient for overall growth, size, and population-level morphological consistency, whereas lumen architecture, branching topology, internal spatial organization, and layer-specific cytoarchitecture require volumetric imaging. Nevertheless, both ends of this imaging spectrum generate analytical challenges: high-throughput 2D imaging produces large numbers of images requiring scalable automated analysis, whereas 3D and multimodal imaging generates increasingly complex and information-rich datasets requiring multidimensional interpretation. This convergence of data scale and analytical complexity creates a broad and growing need for AI-assisted image analysis.

### 2.2 Artificial intelligence: towards comprehensive intelligent analysis

**Fig. 3:**
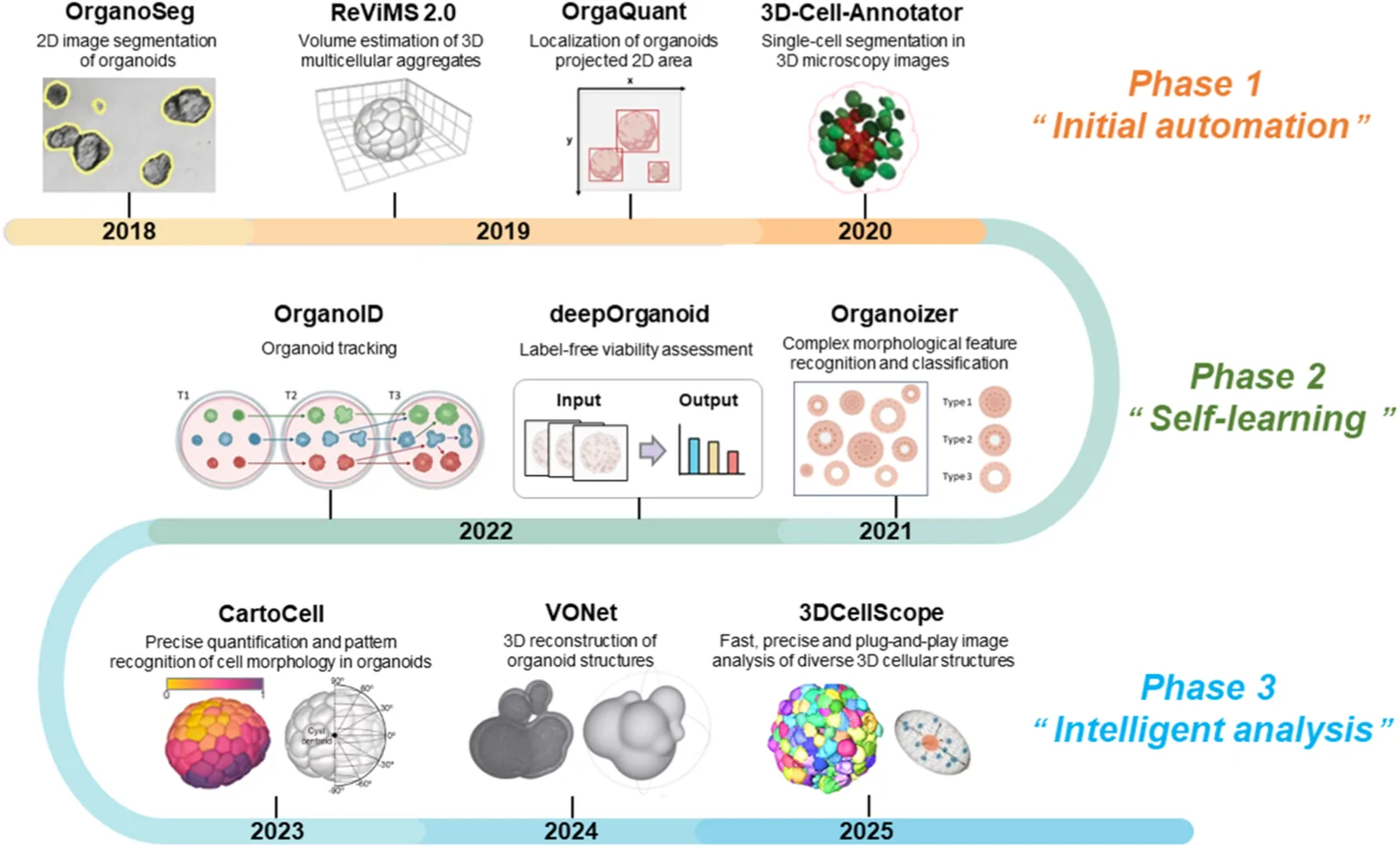
Representative tools and their evolutionary trajectory for organoid morphological analysis. The development is divided into three phases: **Phase 1**: Conventional image processing and early ML methods enabled initial automation of feature extraction from organoid images, exemplified by tools such as OrganoSeg [40], ReViMS 2.0 [41], OrgaQuant [42], and 3D-Cell-Annotator [43]; **Phase 2**: DL-based autonomous analysis tools emerged, represented by OrganoID [33], deepOrganoid [44], and Organoizer [45]; **Phase 3**: Intelligent analytical platforms for comprehensive morphological assessment were developed, including CartoCell [46], VONet [47], and 3DcellScope [48].

The rapid advancement of imaging technologies has generated an unprecedented volume of high-dimensional image data from organoids, concomitantly escalating the complexity of image analysis. Conventional image analysis approaches, characterized by low throughput and inherent subjectivity, have emerged as critical bottlenecks impeding research progress. The integration of AI represents a transformative paradigm shift, converting vast image datasets into objective, quantifiable biological insights [39]. Mirroring the evolutionary trajectory of imaging modalities, AI-driven organoid image analysis has evolved from “initial automation” toward “self-learning” and “intelligent analysis” (Fig. 3).

**Phase 1: Conventional image processing and early ML approaches for initial automation of organoid image analysis.** Early workflows primarily relied on tools based on conventional image processing methods such as ImageJ [49] and CellProfiler [50]. While these tools offered an annotation-efficient workflow by segmenting organoids based on predefined algorithmic rules, they heavily depended on manual parameter tuning [51]. This resulted in low workflow efficiency and restricted cross-dataset generalizability, as the rules were highly sensitive to variations in lighting, background, and organoid types. Furthermore, their applicability was largely restricted to relatively simple 2D imaging data and predefined morphological descriptors, such as area and perimeter. Subsequently, the introduction of early ML models enabled automated feature extraction, allowing detection of subtle morphological changes in organoids [40,42]. However, these methods remained constrained by feature engineering; the requirement for domain experts to manually design features was not only subjective but often failed to capture latent complex patterns, posing significant challenges for high-dimensional data analysis.

**Phase 2: Self-learning approaches enabling autonomous feature extraction.** The emergence of DL has catalyzed a fundamental transformation in organoid image analysis. As a pivotal branch of ML, DL can automatically learn and identify task-relevant features directly from raw images without requiring manual predefinition [2]. This capability significantly improves compatibility with high-dimensional and complex imaging modalities, allowing models to capture the complex nonlinear relationships inherent in organoid developmental processes [9]. However, many standard DL models contain large numbers of trainable parameters and require substantial computational resources and training data, thereby limiting their applicability in resource-constrained scenarios [52]. Notably, certain unsupervised learning approaches exhibit unique advantages. For instance, a voxel-based unsupervised feature learning method, termed “shallow learning”, effectively discovers and interprets meaningful patterns in high-dimensional spaces without requiring manual annotations or prior feature assumptions, providing an effective alternative for high-dimensional analysis when computational resources or labeled data are limited [53].

**Phase 3: Intelligent analysis platforms for comprehensive morphological assessment.** The continuous advancement of AI technology has catalyzed the development of intelligent platforms for organoid morphological analysis. Representative platforms such as CartoCell [46] and 3DcellScope [48] seamlessly integrate the advantages of conventional image processing algorithms with DL techniques, breaking previous modality boundaries to enable precise 3D segmentation of organoid structures and their cellular components, multidimensional feature extraction, and visual representation. These innovations have significantly advanced the field of digital organoids, effectively translating complex biological structures into quantifiable, virtual representations [54]. Meanwhile, the development of generative AI-based image synthesis technologies, exemplified by VONet [47] and related works [55,56], offers a powerful solution to the longstanding annotation bottleneck by automatically synthesizing high-fidelity virtual organoid datasets with ground-truth labels. These advancements signify that organoid morphological analysis is evolving toward greater comprehensiveness and standardization.

As outlined above, the representative analytical methods in OIM exhibit distinct technical profiles, differing significantly in imaging compatibility, annotation dependency, and cross-dataset generalizability. These differences dictate their applicability boundaries and recommended use cases (Table 1). Specifically, rule-based approaches remain useful for standardized images and well-defined morphological endpoints [[49], [50], [51]], whereas standard DL models excel in complex segmentation, classification and prediction tasks when heavily annotated datasets are available [57]. In annotation-limited settings, unsupervised learning approaches can facilitate latent morphological pattern discovery [58]. Meanwhile, integrated 3D analytical platforms are particularly suited to comprehensive structural characterization of organoids, although their transferability depends on imaging standardization and external validation. Ultimately, optimal method selection should be driven by the specific biological objective, data characteristics, and available resources, rather than by model complexity alone.

**Table 1:** Core characteristics and recommended use cases of representative analytical methods for OIM.

Analytical approach	Typical imaging/data compatibility	Annotation dependency	Cross-dataset generalizability	Applicability boundaries	Recommended use cases	Reference
**Conventional image processing and early ML**	Standardized 2D image data	Low to moderate	Low	Sensitivity to imaging variations; limited discovery of latent morphological features	Routine quantification of predefined morphological features; applications requiring transparent and interpretable workflows	[40,42,[49], [50], [51]]
**Standard DL**	High-dimensional images	High	Variable	Limited labeled data or computational resources; stringent interpretability requirements	Complex feature extraction and morphological analysis tasks (segmentation, classification, prediction)	[52,57]
**Unsupervised learning**	High-dimensional image data	Low	Variable	Stringent interpretability requirements	Latent feature discovery with limited labeled data	[53,58]
**Integrated 3D analytical platforms**	High-resolution 3D data	Platform-dependent	Validation-dependent	Limited computational resources; insufficiently standardized imaging data	Comprehensive 3D morphometric analysis and cellular-level structural characterization	[46,48]

**Abbreviations:** 2D, two-dimensional; 3D, three-dimensional; DL, deep learning; ML, machine learning.

The intelligent evolution of AI in organoid image analysis has driven the progressive decoding of complex biological narratives embedded within massive datasets. It is precisely the synergistic advancement and cross-disciplinary integration of AI and imaging technologies that have catalyzed the emergence of OIM.

### 2.3 Synergizing imaging and AI: constructing the operational OIM workflow

The preceding sections have delineated the parallel evolutionary trajectories of imaging technologies and AI. As two foundational pillars of OIM, imaging and AI are coordinated within an analytical workflow that transforms morphological observations into quantitative representations of the organoid morphome and biologically meaningful inferences. The conceptual distinctiveness of OIM does not arise from any individual imaging modality, AI algorithm, or analytical technique. Rather, it lies in organizing these existing and emerging capabilities around the organoid morphome within a structured analytical architecture. In its fully integrated form, the operational architecture of OIM comprises four interconnected phases: (1) standardized morphological data acquisition, (2) AI-driven multidimensional morphome construction, (3) morphology–biology association and predictive modelling, and (4) biological validation.

The first phase establishes standardized and biologically informative imaging data through appropriate spatial, temporal, and multimodal acquisition. AI-driven feature extraction and representation learning then transform these imaging data into multidimensional morphome representations that integrate morphological features with spatial organization and temporal dynamics. These representations can subsequently be linked to predefined developmental, functional, pathological, or treatment-related endpoints to establish associations between organoid morphology and biological states or outcomes and to enable predictive modelling. Where complementary molecular or cellular information is available, multimodal integration can further relate morphological states to underlying molecular or cellular states. Mechanistic interpretations, however, require independent experimental validation. This validation step connects computational inference with biological evidence: supported relationships can refine morphome representations, endpoint definitions, and analytical models, whereas unsupported predictions can expose limitations in data acquisition, representation, or computational inference.

Importantly, existing studies generally implement individual components of this framework rather than the complete OIM workflow within a single experimental system. Therefore, OIM should not be interpreted as a rigid classification criterion requiring existing studies to fulfill all four phases. Instead, it provides a morphology-centered framework for organizing current methodological advances from morphological acquisition and quantification to biological association, prediction, and mechanistic validation, while a fully integrated implementation remains an emerging objective. Accordingly, OIM is best understood as a morphology-centered analytical paradigm that organizes existing and emerging technologies around a defined sequence from morphome construction to biological inference and experimental validation, rather than as a standalone methodological innovation.

## 3 OIM enables a paradigm shift in organoid analysis

The advent of OIM is transforming organoid analysis from an experience-dependent descriptive discipline into a quantifiable, predictive data-driven science. This paradigm shift reshapes how we observe, interpret, and harness morphological information from organoids, operationalized through three critical transitions: from qualitative to quantitative, from static to dynamic, and from superficial to in-depth.

### 3.1 From qualitative to quantitative: establishing an objective foundation for comparative analysis

Before the advent of automated organoid image analysis tools, researchers predominantly relied on manual counting of organoids, as well as on visual inspection of organoid appearance, describing their morphology with terms such as “spheroid” or “cyst” [4]. This qualitative approach is not only labor-intensive and time-consuming but also highly subjective, as different observers may interpret the same image differently, leading to poor reproducibility. Consequently, large-scale comparative studies and high-throughput screening have long been hindered. In response to these challenges, early automation efforts employed rule-based image analysis pipelines, which achieved objective cell/nuclear boundary detection and enabled high-throughput cell counting with enhanced reproducibility [49,59,60]. Nevertheless, these approaches remained constrained by their dependence on hand-crafted features and manual annotation requirements. Moreover, the necessity for fluorescent labeling to enhance visibility introduced additional technical complications, as uneven staining frequently compromised analytical accuracy [61]. These shortcomings underscore the need for more intelligent, label-free solutions.

**Fig. 4:**
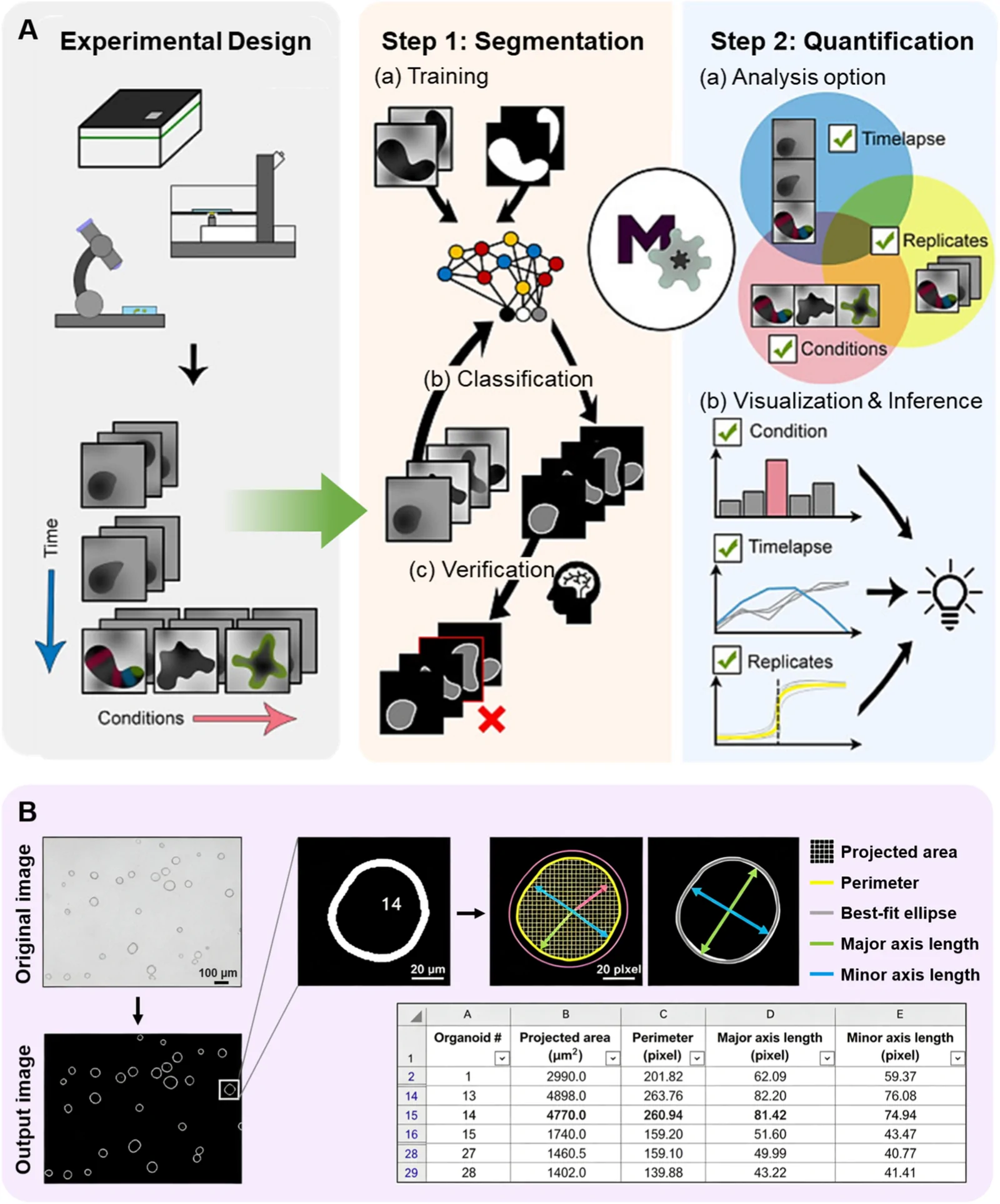
Representative tools for quantitative analysis of organoid morphology. A) MorgAna, enabling image segmentation and quantitative analysis across diverse experimental conditions. Copyright 2021, The Company of Biologists [80]. B) OrgaExtractor, extracting organoid image contours, quantifying diverse morphological metrics, and exporting the quantified data as an Excel-compatible file. Copyright 2023, The Author(s) [16]. These quantitative image-analysis tools constitute the foundational morphological quantification layer of OIM by converting microscopy images into standardized numerical features. Their outputs can subsequently support AI-based classification, functional prediction, dynamic tracking, and biological validation.

Within the OIM framework, DL-based approaches enable precise, objective, and high-throughput quantification of organoid morphology directly from microscopy images, without requiring fluorescent labels. Specifically, after detection [[62], [63], [64], [65], [66]] and segmentation [[67], [68], [69], [70], [71], [72], [73], [74], [75], [76], [77], [78], [79]] of organoids and their internal structures, OIM further enables: **1) Number and size quantification:** OIM automate the counting of individual organoids [16,33,80,81] and their constituent cells [43,46,48,[82], [83], [84], [85]], while simultaneously identifying specialized structures such as lumens in intestinal organoids [86] or trabeculae and chambers in cardiac organoids [87,88]. Beyond simple counting, these systems precisely measure 2D projected areas [42,[89], [90], [91]] or 3D volumes [41], providing robust metrics for organoid size quantification (Fig. 4).

**2) Density and texture analysis:** By calculating ratios such as cell number to area/volume or surface area to volume, OIM quantifies parameters including compactness and solidity, which reflect cellular packing density and tissue organization [33,45,92,93]. Similarly, the volumetric ratio of viral markers to nuclear volume enables precise quantification of viral transduction efficiency in organoids [94], which is a measurement that is difficult to achieve solely through human observation. **3) Shape metric quantification:** OIM transcends human visual limitations through geometric metrics, including circularity, sphericity, shape factor, convexity, jaggedness, and eccentricity [80,93,[95], [96], [97]]. These parameters enable objective morphological classification that captures subtle morphological variations invisible to the human eye, revealing previously unappreciated complexity in organoid development and response.

These quantitative metrics effectively convert visual morphological information into digital data. Equipped with these parameterized features, researchers can compare morphological variations across culture conditions or medicine treatments and establish statistically significant associations. This quantitative foundation serves as the essential prerequisite for the two paradigm shifts discussed in the following sections.

### 3.2 From static to dynamic: restoring the continuity of biological processes

A fundamental biological value of organoids lies in their capacity to faithfully recapitulate the dynamic complexity of *in vivo* tissues *in vitro*, including key processes such as development, regeneration, and pathological progression [98]. Conventional endpoint assays, however, are invasive and terminal, thus incapable of capturing these dynamic processes. In long-term live imaging, organoids and their cells exhibit dynamic spatial arrangements. Traditional analytical approaches, constrained by poor cross-frame association capabilities, cannot track individual targets continuously, leading to the loss of critical dynamic information, such as differentiation trajectories, growth kinetics, and drug response profiles [99,100]. To overcome these challenges, the OIM framework leverages AI-driven time-series analysis and high-throughput data processing to enable multiscale dynamic tracking, spanning from macroscopic tissue architectures down to microscopic cellular entities, thereby reconstructing the temporal continuity of biological processes.

**Fig. 5:**
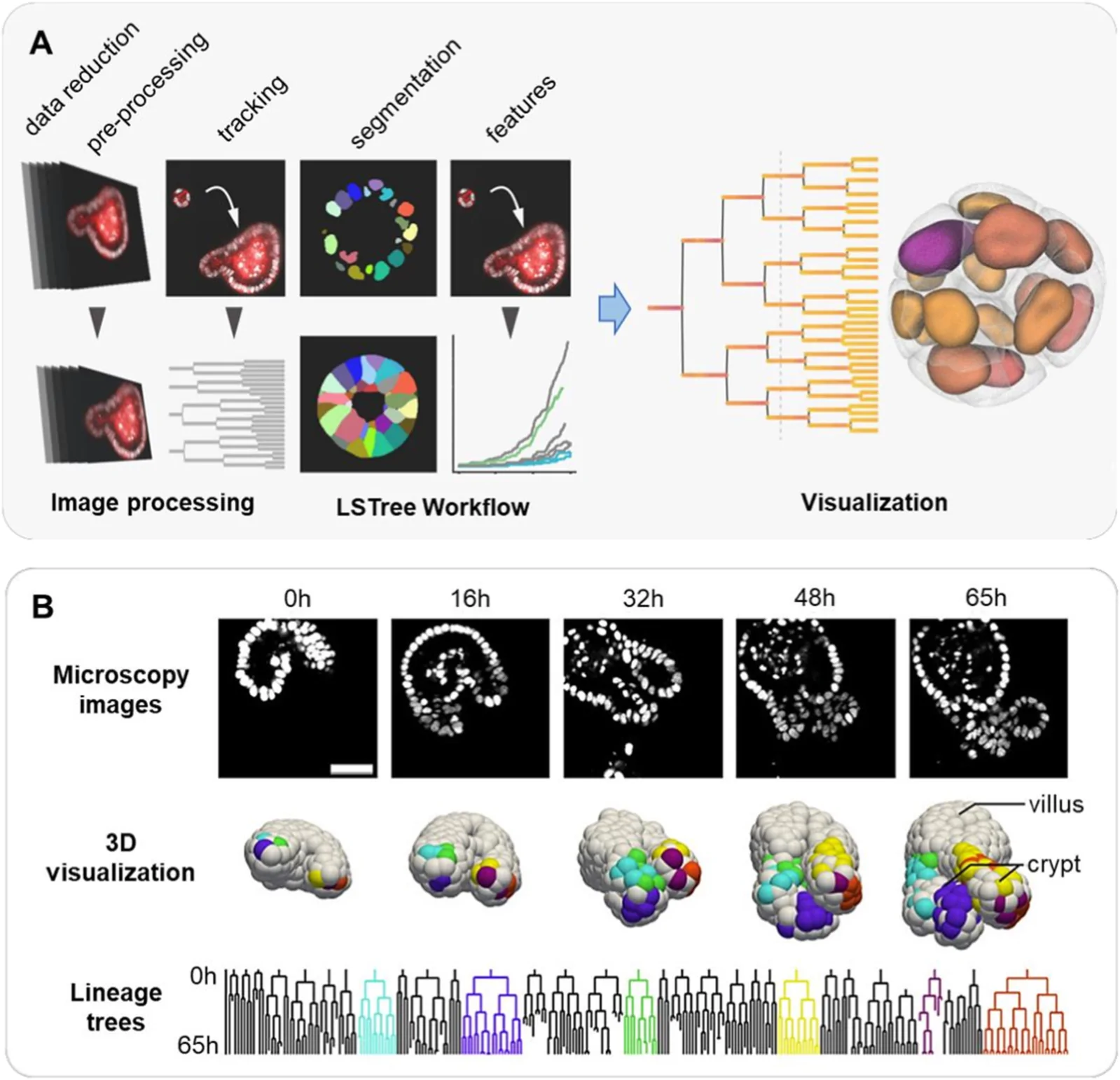
Dynamic tracking in OIM. A) LSTree, a multiscale light-sheet imaging framework, depicting imaging stages, analysis workflow, and visualization tools utilizing dual-fluorescent structural labeling (H2B-mCherry for cell nuclei and mem9-GFP for cell membranes). Copyright 2022, The Author(s) [107]. B) OrganoidTracker, enabling 3D visualization of sequentially captured organoid images, cell tracking via nuclear H2B-mCherry labeling, and algorithm-based lineage tree reconstruction derived from cell division histories. Copyright 2020, Kok et al. [108].

For holistic organoid dynamic monitoring, DL-based tools such as OrganoID [33], OrBITS [101], OrganoIDNet [97], and TransOrga-plus [102] can match the same organoid across sequential time frames. These tools generate growth trajectories and enable label-free, real-time longitudinal tracking of single organoids [51,[103], [104], [105], [106]], thereby allowing researchers to construct quantitative growth curves and identify key morphogenetic events such as division, fusion, and lumen formation. At the single-cell resolution, AI algorithms establish temporal trajectories by matching cell nuclei based on spatial continuity and similar signal characteristics across consecutive time points. OIM tools such as LSTree [107] (Fig. 5A), OrganoidTracker [108] (Fig. 5B), CellPhenTracker [109], and CytoCensus [110], enable precise localization of cell nuclei within 3D organoid reconstructions, construction of individual cellular motion trajectories, and automated quantification of cell cycle, differentiation rate, and apoptosis rate. Notably, OrganoidTracker 2.0, as an upgraded version of OrganoidTracker, integrates DL with statistical physics methodologies to generate trajectories with per-step error probability predictions, significantly improving tracking reliability [111].

Building upon these single-cell tracking capabilities, OIM further extends to the analysis of dynamic interactions between different cellular components within organoid systems. By tracking the motility trajectories and morphological changes of tumor organoids and immune cells, OIM characterizes tumor-immune cell interaction dynamics, including interaction frequency, interaction duration, and spatial heterogeneity [37,112]. Collectively, OIM enables continuous, multiscale monitoring throughout the entire biological process of organoids, facilitating the transition of organoid research from static analysis to longitudinal dynamic monitoring.

### 3.3 From superficial to in-depth: unveiling the biological information behind morphology

While high-resolution imaging technologies have enabled the visualization of intricate morphological features in organoids, significant challenges remain in extracting biologically meaningful information from these complex datasets. Human visual analysis is inherently limited in detecting subtle morphological changes associated with functional states and molecular mechanisms. Furthermore, conventional image processing approaches, which rely on manually designed features, often fail to capture the complex and implicit relationships embedded within the data, thereby limiting their ability to reveal the underlying biological information behind morphology [17]. In contrast, within the OIM framework, AI-based models leverage self-learning capabilities to establish quantitative relationships between organoid morphology and underlying functional or molecular states. This paradigm shift enables the systematic decoding of biological information embedded within morphological features, as demonstrated in the following contexts.

**Fig. 6:**
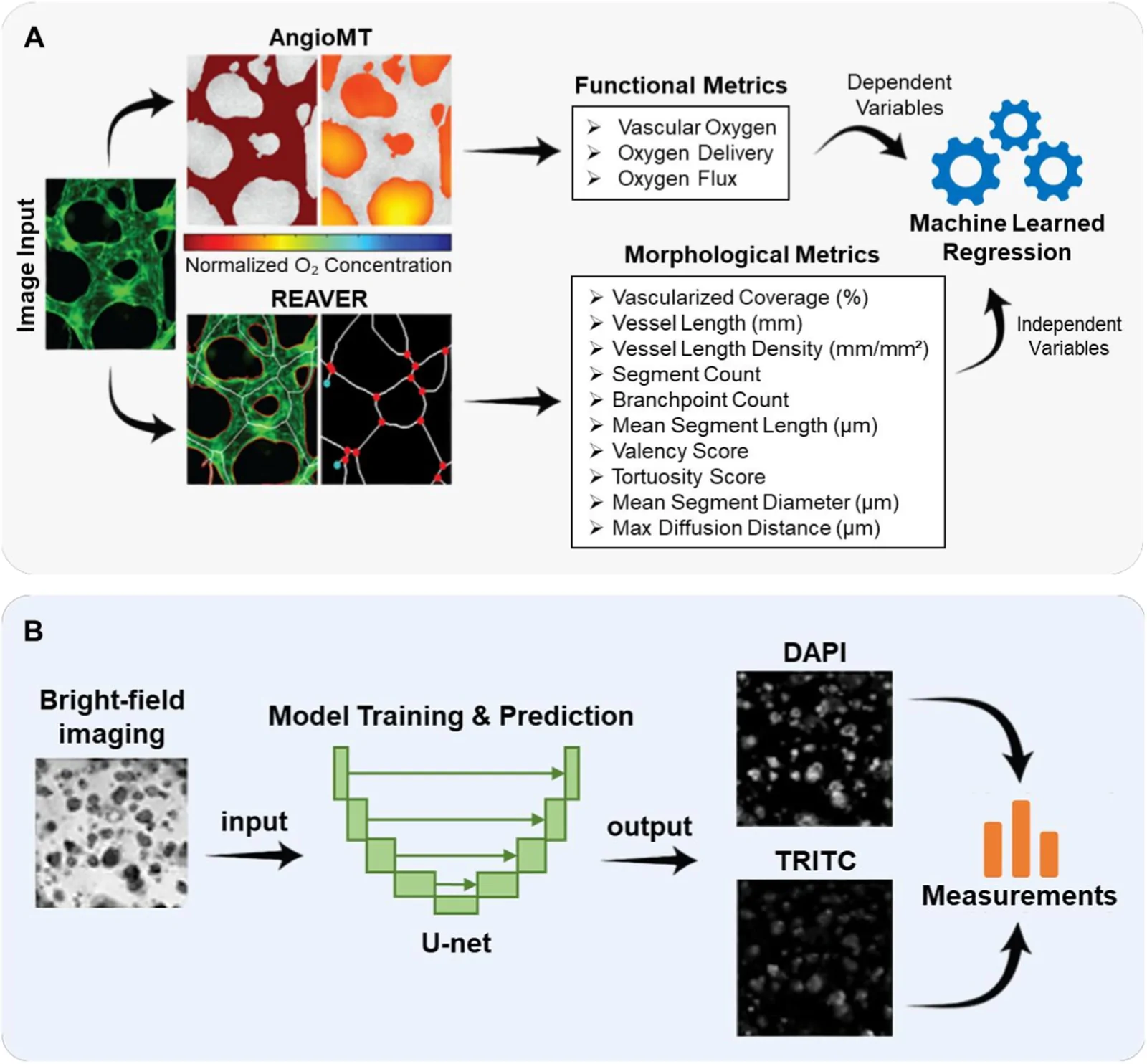
OIM for revealing biological information in organoids. A) AngioMT and REAVER pipelines yielding biological/functional (dependent) and morphological (independent) quantifications for machine learned regression analysis. Copyright 2023, The Author(s) under exclusive license to Biomedical Engineering Society [115]. **B)** DL-based U-Net conversion of brightfield images into fluorescence-equivalent DAPI (nuclei) and TRITC (dead cells) profiles, enabling spatial molecular visualization without physical staining. Copyright 2026, The Author(s) [119].

First, OIM enables the inference of specific biological functions from morphological changes. In vascular network assessment, while early analytical tools could quantify overt morphological metrics such as vessel length, diameter, and coverage [113,114], DL models have advanced this capability by learning the mapping between vascular morphology and oxygen transport function. This allows for the prediction of oxygen delivery potential based solely on morphological features [115] (Fig. 6A). More remarkably, recent DL models can even predict vascular perfusion connectivity from brightfield images without fluorescent beads, enabling comprehensive and non-invasive functional assessment of vascular networks [116]. In addition, OIM can capture biological functions such as secretion [86], differentiation [117], and maturation [87,118] through organoid morphology.

Second, OIM establishes a novel paradigm for integrative morphological–molecular analysis of organoids. AI can classify organoids based on their morphological differences using only label-free microscopic images [[120], [121], [122], [123], [124]]. Subsequent genetic sequencing of these morphologically similar subpopulations then enables the identification of deep genetic drivers underlying morphological variations [105,125]. On this foundation, AI further links organoid morphological changes with molecular shifts (proteomics, transcriptomics, immunofluorescence), forming an integrated morphological–molecular analysis framework. This integration is based on the principle that morphological transformations in organoids typically indicate underlying changes in their cellular composition and function, thereby guiding researchers to investigate associated molecular biomarkers [126]. This integrative approach offers bidirectional analytical capabilities. On one hand, AI can predict the expression levels of organoid biomarkers [[127], [128], [129]] or gene expression profiles [130] from microscopic images, enabling non-destructive detection. On the other hand, AI models can predict not only the expression levels but also the spatial distribution patterns of target molecules within tissues. Using this capability, label-free microscopic images of organoids can be transformed into corresponding simulated fluorescence images, visually presenting molecular spatial expression characteristics without physical staining [119,131,132] (Fig. 6B).

Although these studies leverage AI to explore “morphology–function–mechanism” mappings, they are primarily designed to identify morphological patterns associated with functional or molecular phenotypes rather than to test whether these morphological changes directly cause the observed biological outcomes. This prediction-oriented design reflects both the primary goal of current AI applications and the difficulty of experimentally isolating specific morphological features from the underlying biological processes that generate them. Therefore, AI can reveal predictive relationships from observed data, but cannot by itself establish causality. These associations should consequently be regarded as biologically informative hypotheses that require independent experimental validation to determine their biological significance and potential mechanistic relevance.

In conclusion, OIM has reshaped the methodological paradigm of organoid research by achieving a threefold transformation: from qualitative to quantitative, from static to dynamic, and from superficial to in-depth. This transformation establishes a robust analytical foundation that enables systematic exploration of organoid biology and accelerates the translation of morphological insights into meaningful biomedical discoveries.

## 4 Application of OIM for biomedical discoveries

In this section, we present three major applications of OIM in organoid research, progressing along a translational continuum from ensuring model reliability to mechanistic investigation and ultimately to clinical application: quality control, disease deconstruction, and drug development. These three areas collectively constitute the primary pathway through which OIM facilitates the transition from fundamental laboratory research to translational implementation, as detailed in the following subsections.

### 4.1 Quality control of organoids

Quality control of organoids represents a critical prerequisite for ensuring experimental reproducibility and facilitating clinical translation [133,134]. Based on the temporal dimension of assessment, the application of OIM in quality control can be categorized into two approaches: real-time quality control, which evaluates the current morphological state of organoids, and predictive quality control, which forecasts terminal differentiation outcomes based on early morphological features (Table 2). This dual approach transforms morphological information into quantifiable metrics, thereby establishing a robust foundation for subsequent disease modeling and drug evaluation.

**Table 2:** Applications of OIM in quality control of organoids.

Subcategory	Tool/study	AI task/applications	Organoid type	Imaging modality	Performance	Reference
Viability quantification	Cellos	3D image segmentation for cellular-resolution dynamic analysis	TNBC organoid	Confocal immunofluorescence	F1(IoU = 0.4): 0.853 ± 0.052	[92]
	OrBITS	Semantic segmentation for label-free real-time PDO monitoring	NSCLC organoid	BF	Z′ factor = 0.71–0.8	[101]
	deepOrganoid	Regression analysis for viability mapping	CRC organoid	BF	Pearson r = 0.90	[44]
	EGO-Net	Image segmentation for PCA-derived viability assessment	CRC organoid	OCT	Pearson r = 0.925	[135]
Morphological assessment	HCS-3DX	Imaging segmentation and classification for single-cell 3D screening	3D-oids	LSFM	Accuracy: 98.59%	[136]
	2D-SIGMAT	Image classification for high-precision organoid sorting	CRC organoid	BF and FM	Sorting purity: 80.99 ± 1.25%	[137]
	IMPAT	ML classification for automated morphological sorting	CRC organoid	Inverted microscopy and CLSM	Accuracy >90%	[138]
	3D U-Net + SegFormer	Imaging segmentation and organoid classification	BO	High-field MRI	AUC = 0.98	[139]
	Modified 3	Image classification for vascular abnormality detection	Breast cancer organoid	FM	Accuracy: 90%–99%	[140]
	Trainable Weka Segmentation	Imaging segmentation and quantitative analysis of the tissue structural complexity	Mouse heart organoid	FM	TP rate:0.967–0.999	[87]
	D-CryptO	Image classification for automated scoring of structural maturity	Colon organoid	BF	Accuracy: 90.87%-98%;	[118]
	OrgaSegment	Instance segmentation for CFTR function quantification	PDIO	BF	AP (IoU = 0.5) = 0.76 ± 0.12	[86]
Predictive quality control	EfficientNetV2-S + Vision Transformer	Image classification for predicting secretory function	Hypothalamic-pituitary organoid	BF	Accuracy: 70%	[141]
	VGG19-based model	Binary classification for predicting organoid differentiation	Hypothalamic-pituitary organoid	PC	Accuracy: 79%	[142]
	ResNet50v2	Binary classification for predicting organoid differentiation	Retinal organoid	BF	ROC-AUC: 0.91	[143]
	Ensemble of CNNs	Prediction of tissue formation and size	Retinal organoid	BF	F1 > 0.85	[117]
	ResNet50, DenseNet121, etc.	Regression model for predicting airway differentiation	Airway organoid	BF	R = 0.742–0.812	[127]

***Abbreviations:****DL, deep learning; ML, machine learning; CNN, convolutional neural network; PCA, principal component analysis; PDO, patient-derived organoid; CFTR, cystic fibrosis transmembrane conductance regulator; TNBC, triple-negative breast cancer; NSCLC, non-small cell lung cancer; CRC, colorectal cancer; 3D-oids, 3D models including spheroids, organoids, tumoroids, and assembloids; BO, brain organoid; PDIO, patient-derived intestinal organoid; BF, brightfield microscopy; OCT, optical coherence tomography; LSFM, light-sheet fluorescence microscopy; CLSM, confocal laser scanning microscopy; MRI, magnetic resonance imaging; FM, fluorescence microscopy; PC, phase contrast microscopy; TP, true positive; AUC, area under the curve; ROC, receiver operating characteristic (curve); IoU, intersection over union.*

#### 4.1.1 Real-time quality control

##### 4.1.1.1 Viability quantification

Accurate assessment of organoid viability represents the primary objective of quality control [144,145]. So far, the luminescence ATP-based CellTiter-Glo (CTG) assay has been regarded as the benchmark method for viability assessment. However, this method requires cell lysis, precluding longitudinal tracking of organoid viability dynamics [129,146]. Although existing studies have classified organoid viability based on morphological features in brightfield images [147,148], the accuracy and reproducibility of such approaches remain suboptimal. Consequently, the development of more precise and non-destructive viability quantification methods has emerged as a focal point in current research.

Within the OIM framework, current strategies for non-destructive viability quantification can be broadly classified into three technical approaches: (1) Precise quantification integrated with specific markers: This approach leverages AI to statistically analyze the distribution of fluorescent marker signals for viability assessment. For instance, the Cellos platform employs DL to segment nuclei within organoids, enabling the construction of dose-response curves and the calculation of half-maximal inhibitory concentration (IC50) through counting of viable nucleus [92]. Similarly, the OrBITS platform quantifies the area proportion of dead cell dye signals to directly compute mortality rates and distinguish between cytotoxic and cytostatic effects [101]. (2) End-to-end image-to-viability mapping methods: These approaches train DL models to directly learn complex mapping relationships from raw images to viability values. A representative example is the deepOrganoid model, which predicts cellular viability of organoids based on brightfield images, demonstrating high correlation with the gold-standard ATP luminescence assay (Pearson *r* = 0.90) [44]. (3) Morphological feature extraction with subsequent viability correlation: This strategy first extracts and quantifies morphological features using AI algorithms, then correlates these features with viability metrics. For example, the Trettner team developed and utilized the SAAVY algorithm to calculate viability values of pancreatic ductal adenocarcinoma (PDAC) spheroids by extracting morphological features associated with viability, such as the transparency of the spheroid and the overall morphology, achieving high concordance with human expert judgments in both clear and noisy background conditions [149]. Furthermore, researchers integrated multiple morphological parameters, including volume, surface area, and sphericity, into an aggregated morphological indicator (AMI) through principal component analysis, which exhibited a Pearson correlation coefficient exceeding 0.9 with endpoint ATP measurements [135].

Collectively, these approaches enable continuous, non-invasive monitoring of organoid viability dynamics throughout drug screening processes, thereby overcoming the temporal limitations inherent in conventional endpoint assays.

##### 4.1.1.2 Morphological assessment

Morphological assessment of organoids represents a critical component of quality control [150]. It encompasses three dimensions: (1) population morphological consistency guarantees experimental reproducibility and serves as the prerequisite for statistical comparisons; (2) identification of developmental abnormalities validates model fidelity by enabling the exclusion of compromised specimens; (3) recognition of function-associated structures evaluates physiological relevance and reflects the functional maturation status of organoids. These three dimensions are elaborated below.

**Fig. 7:**
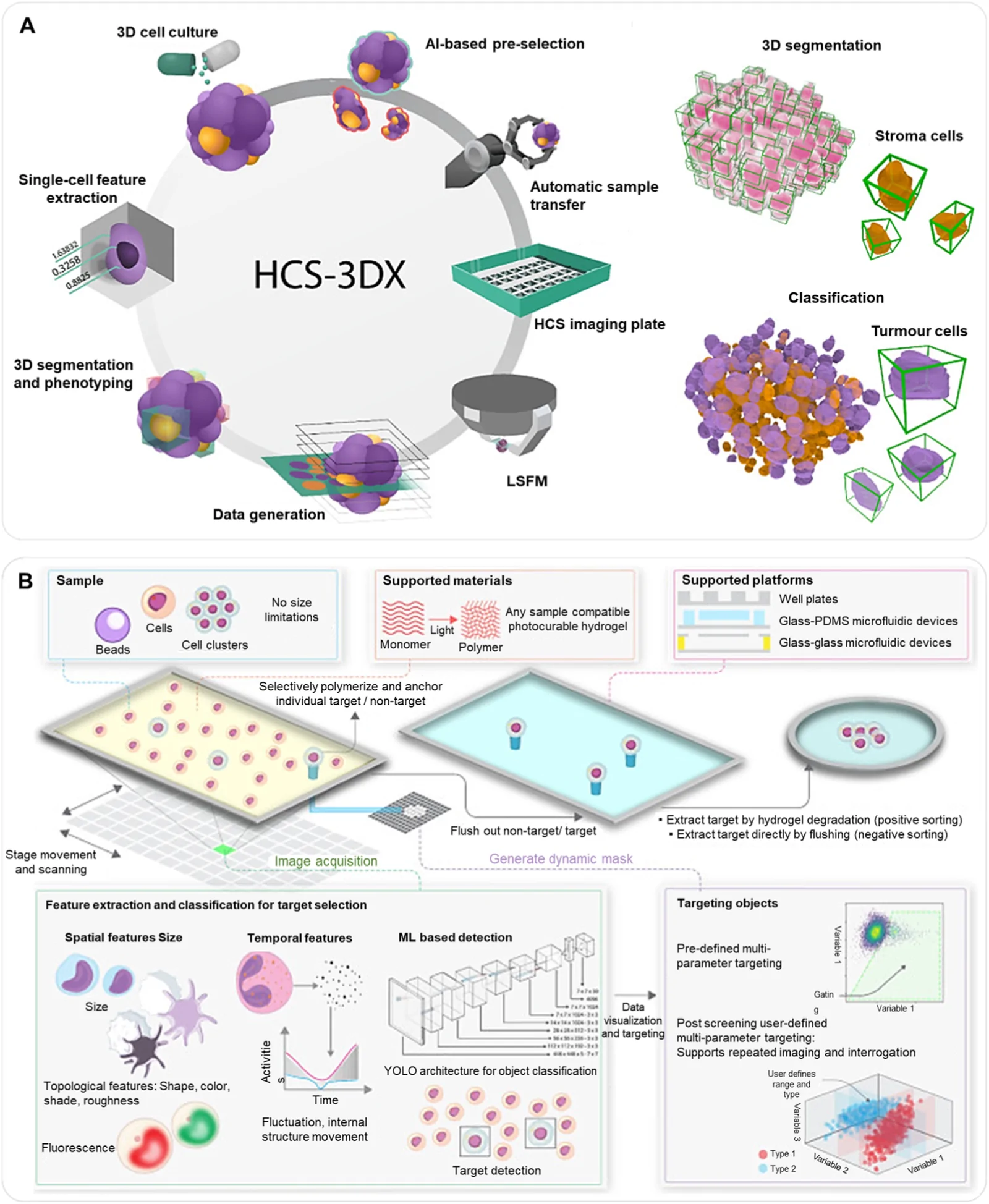
OIM-based intelligent organoid sorting system. A) HCS-3DX platform, integrating AI-guided pre-selection, automated handling, and LSFM-based 3D imaging into a closed-loop workflow for single-cell-resolved morphological profiling of organoids. Copyright 2025, The Author(s) [136]. B) 2D-SIGMAT platform, analyzing high-resolution images via conventional image processing or ML algorithms, for selective target encapsulation and isolation based on spatiotemporal, morphological, or fluorescent features. Copyright 2025, The Author(s) [137].

Variability in size and shape among organoids, both within and across batches, represents a primary source of experimental irreproducibility. OIM addresses this challenge by selecting morphologically uniform individuals from existing cultures, thereby ensuring population-level consistency. For instance, the HCS-3DX system employs DL algorithms to quantify parameters such as diameter, perimeter, area, and sphericity, enabling the selection of morphologically uniform organoids [136] (Fig. 7A). Similarly, the 2D-SIGMAT platform, a novel image-assisted cell sorting system, utilizes DL models to screen for size-consistent colorectal cancer organoids [137] (Fig. 7B). Furthermore, the DOSS system combines droplet microfluidics for generating uniformly sized colorectal cancer organoid units with a ML-based image classification system, achieving real-time detection and phenotypic classification of distinct organoid morphologies (solid, lumen and sparse) [138]. However, the size compatibility of intelligent organoid sorting is governed by an interplay between biological viability and platform geometry, encompassing fluidic channels, droplets, trapping chambers, and optical fields. Although current systems routinely process organoids spanning tens to several hundred micrometers, the absolute upper limit of sortable diameters remains systematically uncharacterized.

In organoid culture, abnormal phenotypes or structures frequently deviate from the normal developmental trajectory. OIM has important applications in detecting such aberrations. For example, to identify phenotypes that deviate from normal development, an ML model based on features including area, circularity, and solidity distinguished normal from defective organoids with an accuracy of ∼0.9 [151]. For abnormal structures during development, such as cyst formation in cerebral organoids, combining high-field magnetic resonance imaging (MRI) with AI enabled automatic discrimination between cystic (low-quality) and non-cystic (high-quality) structures with high accuracy (ROC AUC = 0.98) [139]. Additionally, a DL model has been shown to automatically classify normal and abnormal vasculature in vascularized organoids, demonstrating the feasibility of automated quality assessment [140].

The presence of specific morphological structures often directly correlates with the anticipated physiological functions of organoids. Therefore, precise identification and quantification of these structures serve as critical indicators of functional maturation. In heart organoids, a new ML enables quantitative assessment of myocardial structural maturation by calculating the internal-external perimeter ratio, a parameter that reflects the internal structural complexity of the heart organoids [87]. For colon organoids, the D-CryptO tool employs DL to analyze crypt formation and tissue opacity from brightfield images, providing automated scoring for structural maturation and objective quantification of colonic organoid maturity [118]. For patient-derived intestinal organoids, the OrgaSegment model segments organoids in brightfield images and quantifies CFTR-dependent fluid secretion function by analyzing images from a drug-induced swelling assay, thereby directly correlating lumen size with physiological function [86]. Collectively, these examples illustrate how OIM transforms structural features into quantifiable readouts of organoid functionality.

#### 4.1.2 Predictive quality control

The ultimate differentiation outcome of organoids is linked to morphological changes occurring during their early developmental stages. Critical morphogenetic events, such as lumen formation and structural polarization, serve as predictive indicators of subsequent cellular composition and functional differentiation trajectories. Within the OIM framework, DL-based predictive models can analyze brightfield images acquired during early differentiation to establish associations between early morphological features and late-stage cellular composition or secretory function. This paradigm shift enables substantial advancement of quality control checkpoints, thereby minimizing the costs associated with futile late-stage cultures.

**Fig. 8:**
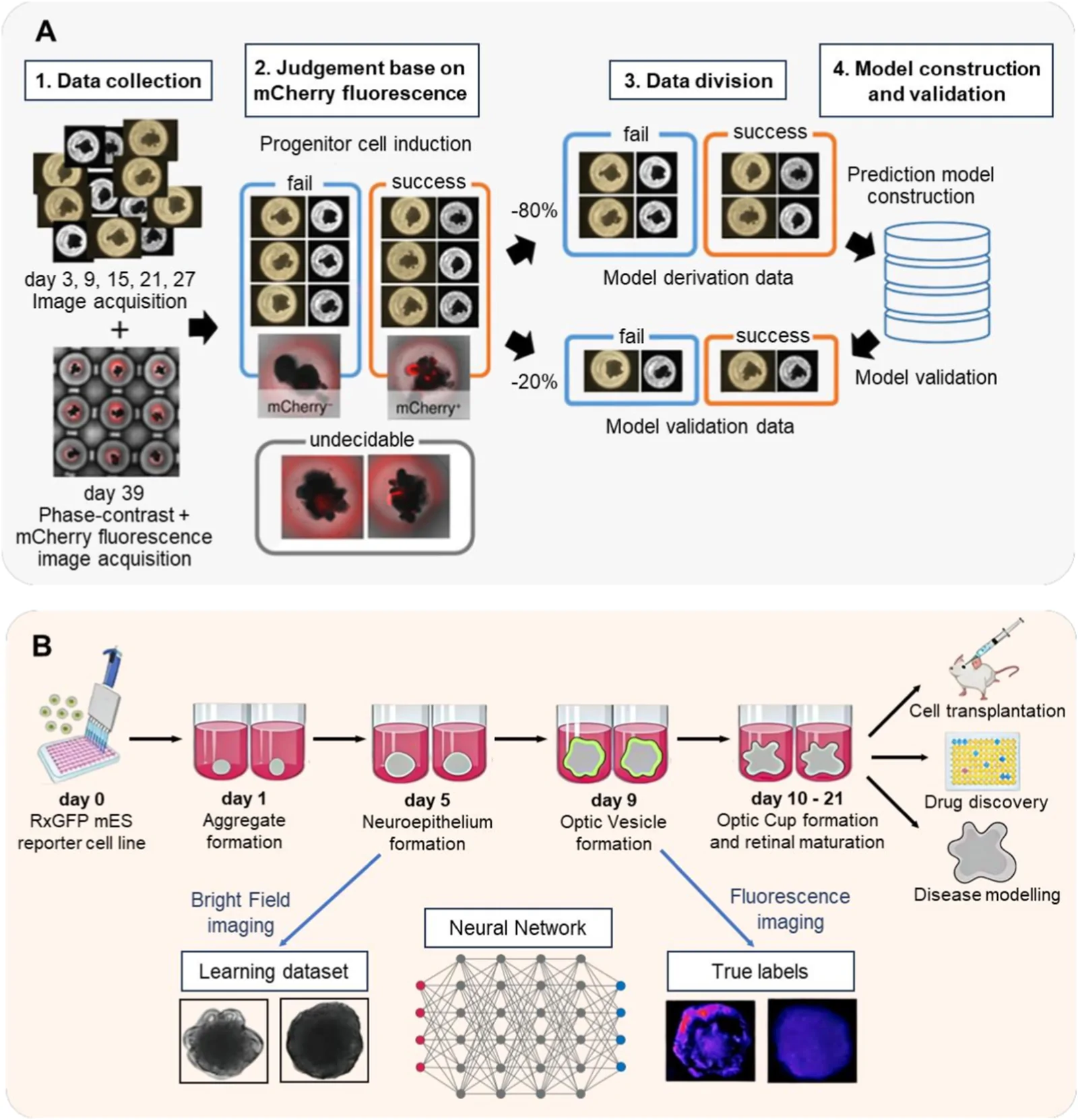
Representative approaches for predicting organoid developmental and differentiation trajectories within the OIM framework. A) Workflow for prediction model development, encompassing data collection, mCherry fluorescence-based judgment, data division, model construction, and validation. Copyright 2025, The Author(s) [142]. B) Neural network training utilizing brightfield microscopy (day 5) and fluorescence staining of early retinal markers Rx/Pax6 (day 9) to predict early retinal differentiation, with fluorescence images serving as the reference standard. Copyright 2024, The Author(s) [143].

Recent AI-based studies encompassed by the OIM framework have progressively shifted the prediction of organoid differentiation toward earlier developmental stages. This progression is exemplified by pituitary organoids, which demonstrate robust predictive performance: initial studies achieved 70% accuracy in predicting final secretory function based on day 30 images [141]. Notably, subsequent algorithmic optimizations advanced the prediction time point to as early as day 9 of differentiation, while maintaining 79% accuracy in forecasting differentiation outcomes at day 40 [142] (Fig. 8A). This earlier prediction capability provides researchers with substantially longer timeframes to adjust culture conditions or implement pharmacological interventions, thereby markedly enhancing experimental efficiency and success rates. The robustness of this predictive strategy has been validated across diverse organoid systems, including hypothalamic-pituitary organoids [141], retinal organoids [143,152], airway organoids [127], kidney organoids [128], and ovarian cancer organoids [153]. Collectively, these applications demonstrate the versatility of OIM-based predictive quality control in reducing experimental timelines and optimizing resource allocation.

Beyond advances in prediction timing, the datasets supporting morphology-based predictive quality control have expanded substantially in scale, providing a stronger foundation for model development and validation. This progression is exemplified by retinal organoid research, which has progressed from limited-sample binary classification (retinal vs. non-retinal organoids) to the use of comprehensive large-scale datasets [143] (Fig. 8B). The current state-of-the-art dataset comprises approximately 1000 organoids and over 117 000 time-series images, representing an order-of-magnitude increase in organoid numbers and a more than 100-fold expansion in image volume compared to earlier studies [117]. This dramatic increase in data scale provides a significantly more robust foundation for training predictive models based on early morphological features, thereby improving prediction reliability and enhancing generalizability across diverse organoid platforms.

### 4.2 Disease deconstruction

Having established a standardized foundation for organoid research through rigorous quality control, OIM has been further leveraged for disease deconstruction, systematically linking morphological phenotypes to their underlying biological mechanisms (Table 3).

**Table 3:** Applications of OIM in disease deconstruction.

Subcategory	Tool/study	AI task/applications	Organoid type	Imaging modality	Performance	Reference
Quantitative phenotyping of disease morphology	SCOUT	Image segmentation and classification for cellular, spatial, and tissue feature quantification	Human cerebral organoid	LSFM	Dice coefficient: 0.972	[36]
	OrganoIDNet	Image segmentation and classification for patient-specific therapy response profiling	PDAC organoid	BF	IoU = 0.74	[97]
	Integrated workflow	Image segmentation and classification for PDO growth dynamics analysis	CRC organoid	BF	Mean normalized fitting error: 2.01%–10.11%	[96]
Linking morphological phenotypes to disease mechanisms	Dual DNN framework	Classification and regression for image-to-transcriptome feature mapping	PDTO	BF	Pearson r = 0.78	[130]
	AI-based classifier	Image classification for linking morphology to drug sensitivity	CRC organoid	BF	Accuracy: 72.4%	[154]
	Segment Anything Model (v1.0)	Organoid morphology-guided classification	OSCC organoid	DIC	Classification accuracy: 82%	[93]

**Abbreviations:** DNN, deep neural network; PDO, patient-derived organoid; PDAC organoid, pancreatic ductal adenocarcinoma organoid; CRC organoid, colorectal cancer organoid; PDTO, patient-derived tumor organoid; OSCC organoid, oral squamous cell carcinoma organoid; LSFM, light-sheet fluorescence microscopy; BF, brightfield microscopy; DIC, differential interference contrast microscopy.

#### 4.2.1 Quantitative phenotyping of disease morphology

OIM has emerged as an objective, high-throughput, and non-destructive platform that enables longitudinal tracking of disease progression. By automating the extraction of morphological features from organoid images, OIM enables quantitative assessment of morphological dynamics across diverse disease models, thereby enabling comprehensive pathological characterization.

**Fig. 9:**
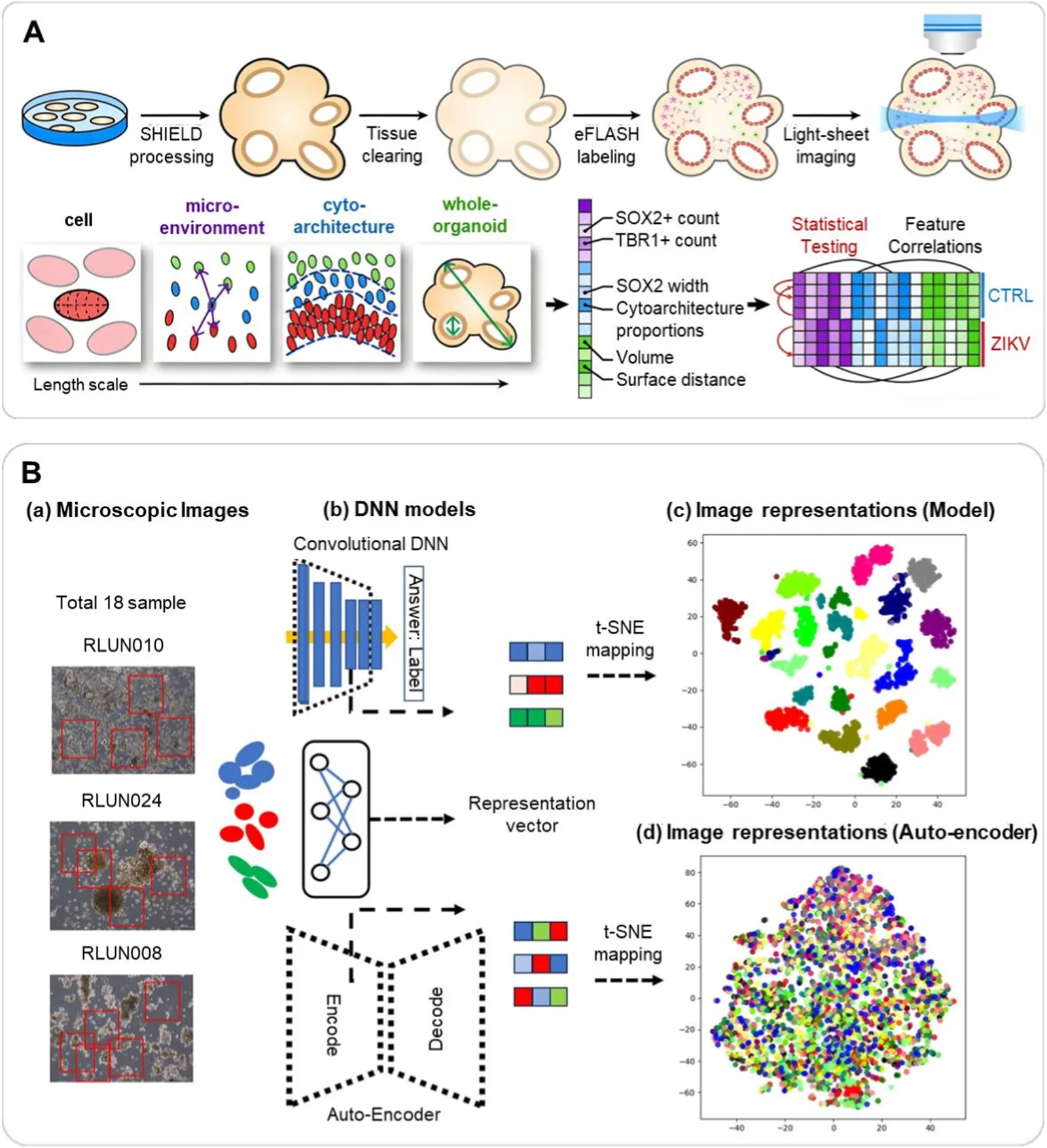
Quantitative analysis and prediction of organoid disease models using OIM**.** A) Multiscale hyperdimensional analysis pipeline, leveraging automated algorithms and convolutional neural networks for quantitative multiscale feature extraction and analysis following organoid culture and imaging. Copyright 2020, The Author(s) [36]. B) DL framework for tumor heterogeneity characterization using PDOs, employing a first deep neural network (DNN) to distinguish original tissue types from microscopy images and a second DNN to predict gene expression from extracted image features to identify inter-tissue differences. Copyright 2024, The Author(s) [130].

A paradigmatic application of OIM is demonstrated by the Single-cell and Cytoarchitecture analysis of Organoids using the Unbiased Techniques (SCOUT) workflow [36]. This DL-powered approach analyzes light-sheet microscopy images to perform multiscale phenotypic profiling of human cerebral organoids. SCOUT quantitatively delineated Zika virus-induced pathological changes, revealing significant reductions in cellularity, disrupted tissue architecture, diminished ventricular volume, and morphological abnormalities [36] (Fig. 9A). In the context of tumor organoids, morphological metrics such as size provide quantitative readouts for chemotherapeutic response assessment, whereas eccentricity serves as a robust indicator of invasive capacity [97]. Moreover, longitudinal tracking of tumor organoid size (area) and morphology (shape factor, sphericity, convexity), integrated with mathematical modeling to derive growth kinetics such as initial growth rate and carrying capacity, offers innovative perspectives for pathological characterization of neoplastic diseases [96].

#### 4.2.2 Linking morphological phenotypes to disease mechanisms

Beyond quantitative phenotyping of disease morphology, OIM enables the use of morphological phenotypes to investigate disease mechanisms, yielding deep biological insights into pathogenesis.

A landmark dual deep neural network framework established a direct mapping between morphological features and transcriptomic signatures in patient-derived organoids (PDOs), revealing enriched pathways associated with phagosome, cell adhesion, and transcriptional misregulation in cancer. This computational approach identified key genes implicated in cancer progression and immune evasion, thereby elucidating potential molecular mechanisms underlying tumorigenesis [130] (Fig. 9B).

Furthermore, OIM enables disease subtyping and mechanistic insights. An AI-based classifier stratified PDOs into distinct morphological subtypes, revealing subtype-specific molecular signatures [154]. Similarly, AI-based morphological analysis of oral squamous cell carcinoma (OSCC) organoids identified three distinct morphological subtypes. Integrated genomic and transcriptomic profiling revealed subtype-specific molecular signatures, and these transcriptomic features demonstrated significant association with clinical prognosis in The Cancer Genome Atlas (TCGA) OSCC patient cohorts [93]. These findings demonstrate that AI-based morphological analysis bridges phenotypic classification with molecular mechanisms, uncovering deeper biological insights into disease pathogenesis.

Therefore, the application of OIM in disease deconstruction can be summarized into two interconnected aspects: (1) quantitative characterization of morphological alterations, and (2) reverse inference of pathological mechanisms in molecular pathways based on morphological features. This approach not only offers new perspectives for future high-throughput, non-invasive investigation of disease mechanisms, but also has substantial significance for guiding patient clinical outcomes.

### 4.3 Drug development

In the preceding section, OIM-based disease phenotyping demonstrated substantial potential in elucidating disease mechanisms. Building upon this foundation, this section extends the application of OIM to drug development, bridging fundamental disease mechanisms research with clinical translation. This section delineates three hierarchical applications of OIM across the drug development continuum: drug screening, drug toxicity assessment, and personalized therapeutic guidance (Table 4), as elaborated in the following subsections.

**Table 4:** Applications of OIM in drug development.

Subcategory	Tool/study	AI task/applications	Organoid type	Imaging modality	Performance	Reference
Drug Screening	LABKIT + IMARIS	Image segmentation for nephron morphometry-based compound screening	Kidney organoid	CLSM	p = 0.019	[155]
	POST	Instance segmentation for growth parameter extraction in pre-screening	Pancreatic and lung cancer organoid	BF	AP: 0.78–0.89	[156]
	CNN	Image classification for phenotype classification and drug effect quantification	Neural organoid	Confocal immunofluorescence	Z′ factor ≈ 1	[157]
Drug toxicity assessment	DILITracer	Ternary classification for spatiotemporal DILI level prediction	Human liver organoid	BF	Accuracy: 82.34%	[158]
	U-Net + AlexNet	Image segmentation and classification for toxicity assessment via area monitoring	Kidney organoid	BF	Accuracy > 95%	[159]
	Random forest classifier	Image classification and prediction for neurotoxicity identification	Human midbrain organoid	CLSM	Accuracy: 86%	[160]
	ImPheNet	Image classification and prediction for phenotype distinction and toxicity assessment	BO	CLSM	Accuracy: 73.2%–96.7%	[161]
Personalized Medication Guidance	Orbits	Organoid tracking and classification of drug sensitivity of organoids	PDAC organoid	BF	Spearman r: 0.9643	[162]
	U-Net + TrackMate (v7.6.1)	Image segmentation and classification for label-free sensitivity testing	Breast cancer organoid	QPI	Jaccard index: 0.897 ± 0.109	[163]

**Abbreviations:** DL, deep learning; ML, machine learning; NDR, normalized drug response; BO, brain organoid; PDAC organoid, pancreatic ductal adenocarcinoma organoid; PDO, patient-derived organoid; CLSM, confocal laser scanning microscopy; BF, brightfield microscopy; QPI, quantitative phase imaging; AP, average precision.

#### 4.3.1 Drug screening

Conventional target-centric drug screening approaches, constrained by predefined molecular targets, exhibit limited capacity to identify compounds with unprecedented mechanisms of action. In contrast, phenotypic drug screening (PDS) circumvents this limitation by directly utilizing cellular or tissue-level phenotypic alterations as screening endpoints, thereby enhancing the discovery potential of first-in-class therapeutics [164]. By enabling the quantitative tracking and analysis of organoid morphological changes before and after drug exposure, OIM provides a range of computational capabilities that can be leveraged for PDS, facilitating objective evaluation of therapeutic efficacy.

**Fig. 10:**
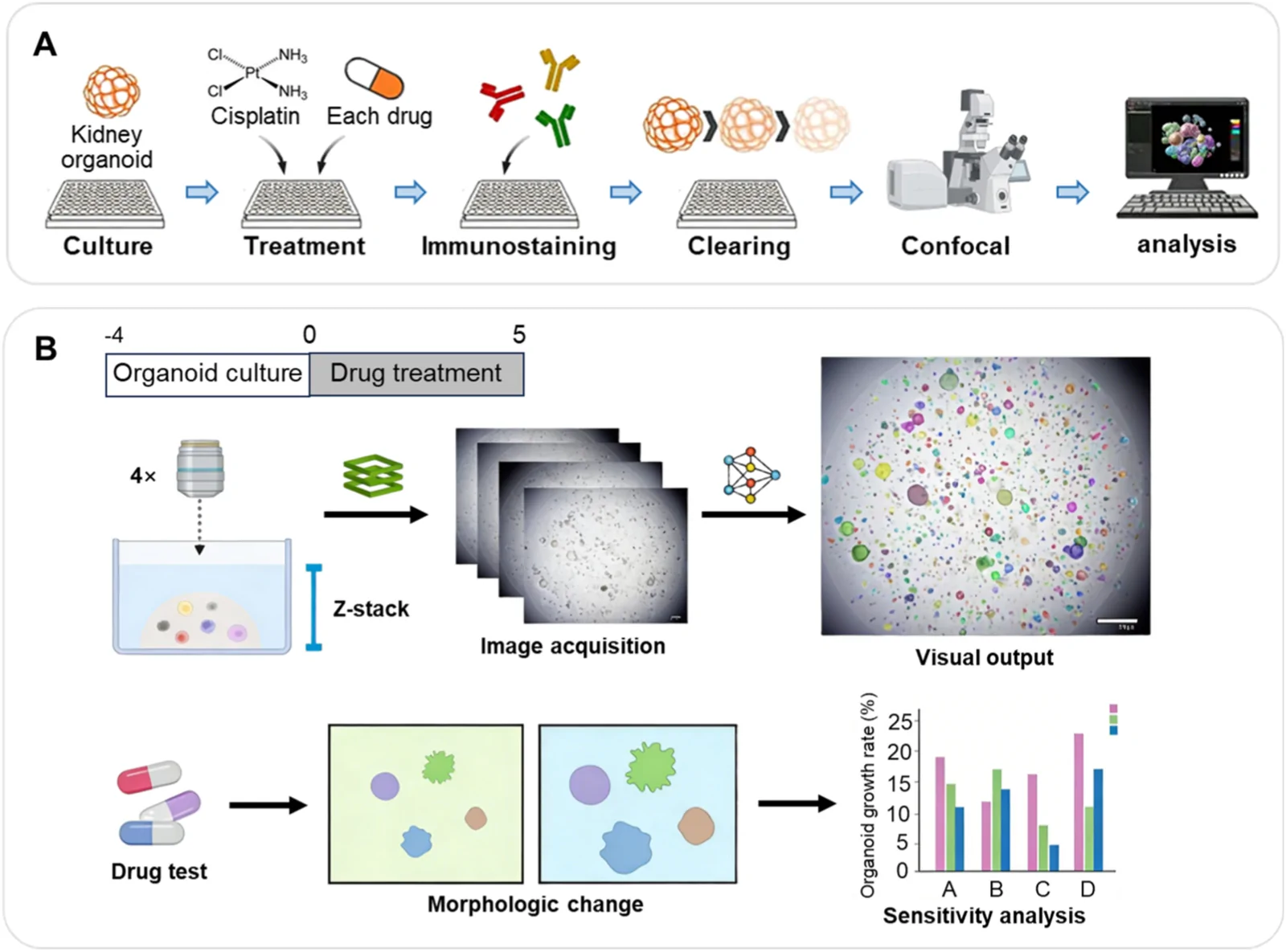
OIM for drug screening. A) Kidney organoid processing workflow encompassing cultivation, drug treatment, immunofluorescence, tissue clearing, and confocal imaging, followed by DL-based 3D morphometric analysis to objectively assess therapeutic efficacy. Copyright 2025, Institute of Physics (Great Britain) [155]. B) DL algorithm POST, automatically segmenting organoids in brightfield images and quantifying morphological features such as growth rate for each organoid, enabling drug sensitivity analysis. Copyright 2025, Xuan Du et al. [156].

In a seminal study, kidney organoids were deployed to screen the FDA-approved drug library (Tocriscreen), where quantitative extraction of nephron segment parameters, including volume, cell number, and spatial density, revealed that imatinib markedly attenuated cisplatin-induced tubular damage, highlighting its potential renoprotective properties [155] (Fig. 10A). Similarly, the precision organoid segmentation technique (POST) quantifies organoid morphological parameters such as growth rate, providing a reliable indicator for assessing antitumor therapeutic efficacy [156] (Fig. 10B). In a Huntington's disease (HD) model, researchers combined DL analysis of wild-type (WT) and HD organoid phenotypes to quantify drug efficacy by assessing the similarity of the sample phenotypes to WT. This approach enabled the identification of bromodomain inhibitors capable of rescuing disease phenotypes from a library of over one thousand compounds [157]. Together, these case studies underscore OIM's transformative potential in PDS. Although large-scale OIM-driven screening campaigns remain nascent, converging advances in automated organoid culture systems, HCI infrastructure, and AI analytics are rapidly maturing. Ultimately, this technological convergence represents a paradigmatic shift toward more predictive, human-relevant drug development platforms that bridge the translational gap between *in vitro* models and clinical outcomes.

#### 4.3.2 Drug toxicity assessment

Drug safety concerns constitute a leading cause of clinical attrition. Conventional toxicity assessment methodologies predominantly rely on animal models or 2D cell cultures, both of which exhibit fundamental limitations: the former is plagued by interspecies translatability issues, whereas the latter fails to recapitulate the complex organ-specific microenvironment. Organoids derived from normal tissues, such as hepatic, renal, cardiac, and cerebral organoids, offer a transformative alternative by faithfully recapitulating key structural architectures and functional attributes of native organs *in vitro*, thereby establishing a more physiologically relevant and predictive platform for preclinical toxicity evaluation. Within the OIM framework, AI-assisted morphological analysis enables non-invasive, real-time detection of subtle morphological perturbations in organoids, facilitating early-stage identification of drug-induced toxicities before irreversible damage occurs.

**Fig. 11:**
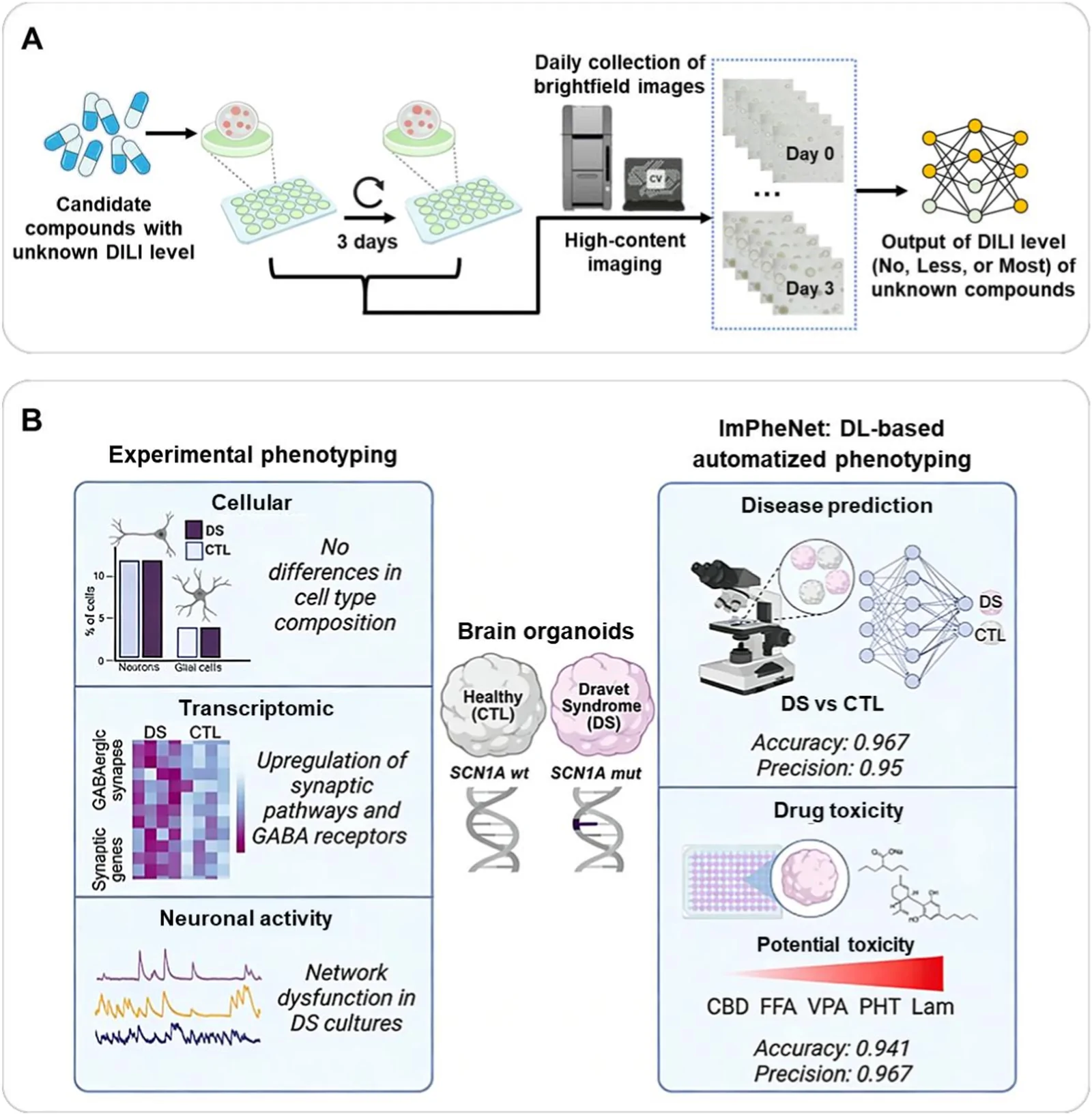
OIM for toxicity assessment. A) AI model for predicting drug-induced liver injury (DILI) levels of compounds with unknown hepatotoxicity. Copyright 2025, The Author(s) [158]. B) DL-based ImPheNet framework building upon experimental phenotyping to enable automated organoid phenotyping, thereby achieving drug toxicity prediction for anti-seizure drugs (VPA, CBD, FFA, PHT, Lam). Copyright 2025, Turpin-Moreno et al. [161].

Drug-induced liver injury (DILI) represents one of the predominant causes of both preclinical attrition and post-marketing drug withdrawal. The DILITracer system employed brightfield imaging of hepatic organoids to automatically classify compounds into three DILIrank categories (vMost-, vLess-, and vNo-DILI concern) with an accuracy of 82.34%, thereby demonstrating the feasibility of non-invasive brightfield imaging for early hepatotoxicity prediction [158] (Fig. 11A). In nephrotoxicity assessment, one study used ML to classify organoids from brightfield images into well-developed, failed, and adhesive categories, quantifying nephrotoxicity by tracking changes in category proportion and per-organoid area [159]. Separately, fully automated 3D imaging integrated with ML quantified the volumetric loss of tubular structures, establishing a robust quantitative metric for tubular injury evaluation [155]. In the field of neurotoxicity, ML enables the prediction of neurotoxic status in human midbrain organoids through the quantification of dopaminergic neuron morphological features, achieving 86% classification accuracy [160]. In another study, multimodal analysis combining live imaging of brain organoids with DL overcame organoid heterogeneity, accurately discriminated disease phenotypes, and predicted the toxicity of antiepileptic agents, offering a powerful platform for neurological drug safety evaluation [161] (Fig. 11B). Collectively, these studies demonstrate that OIM-based toxicity assessment provides non-invasive and quantitative endpoints across multiple organ systems, advancing preclinical safety pharmacology.

#### 4.3.3 Personalized therapeutic guidance

Inter-individual variability in drug response represents a persistent challenge in clinical practice, where identical therapeutic regimens often yield divergent outcomes across patients. Compounding this complexity, intra-tumoral heterogeneity frequently manifests as coexisting cellular subpopulations with distinct drug sensitivities within the same patient. OIM provides a framework for unbiased, quantitative assessment of drug effects across diverse organoid models, thereby facilitating precision medicine approaches and personalized therapeutic decision-making.

Morphological analysis of drug-treated organoids derived from different patients enables quantitative comparison of patient-specific response patterns. Recent studies have demonstrated the utility of OIM in quantifying treatment responses across patient cohorts. For instance, systematic quantification of volume changes in colorectal cancer organoids from different patients under trastuzumab treatment enabled stratification of organoid drug-response phenotypes across patients [135]. Similarly, atezolizumab treatment of organoids derived from different PDAC patients revealed heterogeneous morphological changes, including changes in eccentricity, area, and size, that correlated with inter-patient variability in *in vitro* treatment response [97]. Furthermore, by establishing eight pancreatic cancer PDO lines from distinct origins and utilizing a normalized drug response (NDR) metric, researchers successfully detected drug-resistant, sensitive, and highly sensitive subclonal populations, thereby quantifying their differential responses to therapeutic regimens [162]. These approaches provide a quantitative framework for preclinical drug screening and personalized treatment selection.

Beyond inter-patient variability, OIM also facilitates the identification of drug-sensitive subpopulations within individual patients, enabling precise targeting of specific cellular subsets. A compelling example emerges from OSCC research, where morphological subtypes of PDOs revealed subtype-specific drug responses: Nutlin-3a demonstrated selective efficacy against normal-like OSCC organoids, while dense and grape-like subtypes exhibited intrinsic resistance; notably, combination therapy with an ATR inhibitor and cisplatin proved particularly effective against the dense-type PDOs [93]. Similarly, colorectal cancer organoids were classified into three morphological subtypes (solid, lumen, and sparse) with subsequent drug sensitivity assays revealing that different morphologies exhibited distinct IC50 values when exposed to the same therapeutic agent [138]. ML algorithms tracking mass changes in breast cancer organoids identified distinct subpopulations exhibiting sensitivity, resistance, or stable mass maintenance [163]. These findings underscore OIM's potential to guide combination therapy strategies by simultaneously targeting multiple morphological subtypes or sequentially addressing resistant populations.

In summary, OIM is systematically transforming drug discovery across three critical dimensions: drug screening, toxicity assessment, and personalized therapeutic guidance. Its fundamental value lies in converting morphological features from subjective qualitative endpoints into quantifiable, predictable, and screenable biological parameters, thereby substantially enhancing the efficiency, objectivity, and clinical relevance of the drug development pipeline.

## 5 Challenges

**Fig. 12:**
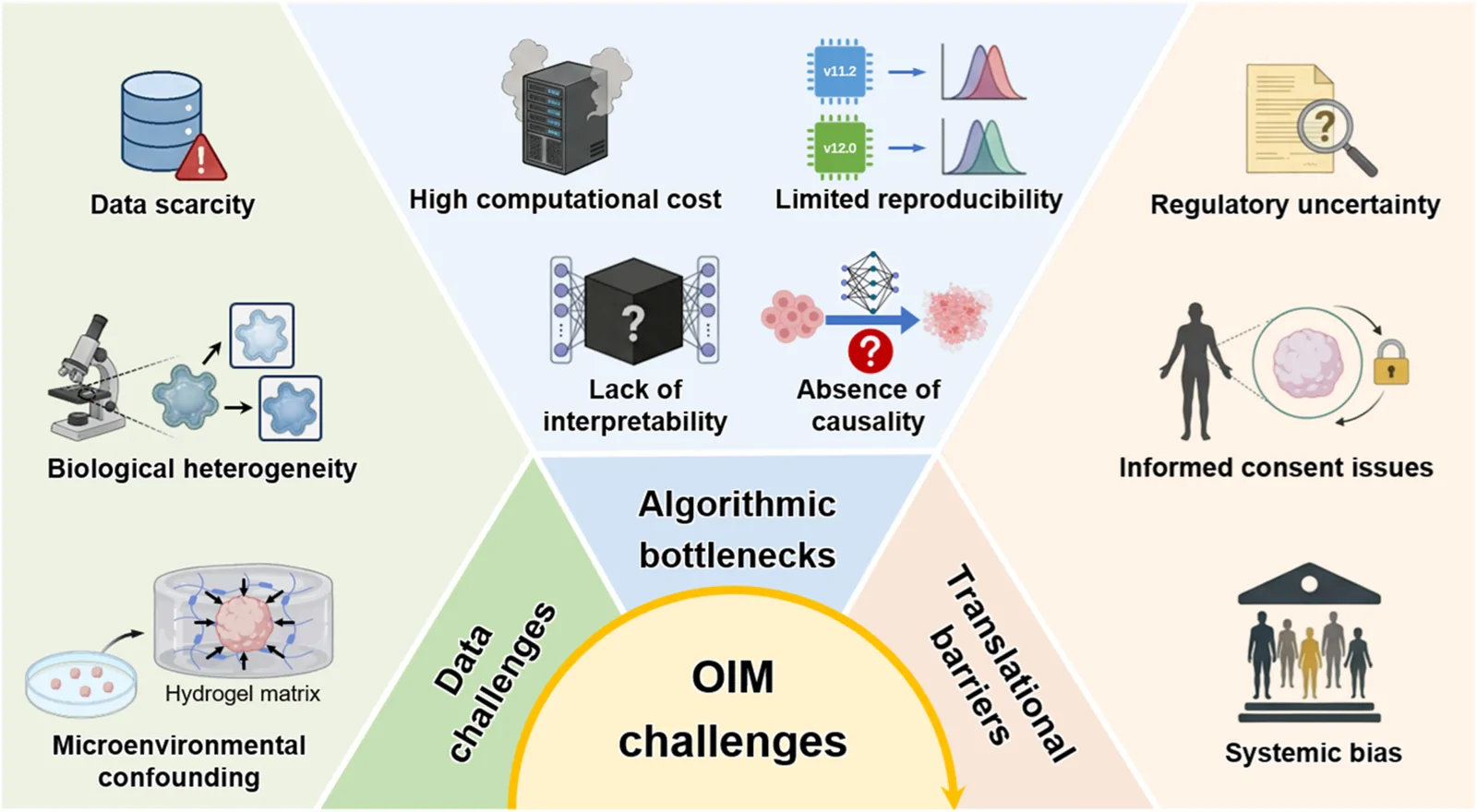
**Key challenges limiting the advancement of OIM.** The development and translation of OIM are constrained by three major categories of challenges: (1) data challenges, including data scarity, biological and technical heterogeneity, and microenvironmental confounding; (2) algorithmic challenges, including high computational cost, limited reproducibility, insufficient interpretability and causality; and (3) translational barriers, including regulatory uncertainty, ethical considerations and systemic bias.

Although OIM has established a framework for transforming organoid morphology into biologically meaningful readouts, its transition from laboratory-scale demonstrations to broadly applicable research and clinical platforms remains constrained by several fundamental challenges. These limitations arise from the scarcity and heterogeneity of organoid imaging data, the difficulty of distinguishing biologically meaningful morphological variation from technical and microenvironmental influences, limitations in computational robustness and mechanistic interpretability, and unresolved barriers to clinical and industrial translation (Fig. 12).

### 5.1 Data challenges: scarcity, heterogeneity, and microenvironmental confounding

The robust deployment of OIM depends on imaging datasets that are sufficiently large, standardized, and biologically contextualized. However, current organoid imaging datasets remain constrained by data scarcity, substantial biological and technical heterogeneity, and incompletely characterized microenvironmental influences, all of which can compromise the reliability of morphology-based analysis.

Foremost among these challenges is the scarcity of high-quality, comprehensively annotated data. Organoid culture is resource-intensive and often exhibits variable experimental success, making large-scale data generation difficult [165,166]. Moreover, the irregular geometry and variable thickness of organoids can introduce technical artifacts during imaging, including local defocusing, light scattering, and signal occlusion [26,33]. These limitations reduce image quality and may impair the reliability of downstream morphological feature extraction, further restricting the availability of high-quality datasets suitable for AI model development.

Beyond data scarcity, substantial biological and technical heterogeneity creates pronounced distribution shifts across OIM datasets. Organoids exhibit intrinsic variability in morphology, cellular composition, and developmental trajectories [167,168]. At the same time, differences in culture protocols, imaging systems, acquisition parameters, preprocessing procedures, and annotation conventions introduce additional technical variation [[169], [170], [171]]. Consequently, a key challenge for AI models is to distinguish biologically relevant morphological variation from systematic variation arising from platform- and experiment-specific factors, particularly when integrating datasets across laboratories and experimental platforms.

Crucially, organoid morphology is jointly shaped by intrinsic biological states and extrinsic microenvironmental cues, creating potential confounding in morphology-based inference. Biophysical and biochemical factors, including matrix micro/nanostructure, stiffness, fluidic forces, mechanical stretch, cytokines, and growth factors, can substantially influence organoid architecture and function by shaping cellular organization and interactions within 3D space [172]. Among these factors, biomaterial properties, such as matrix stiffness, hydrogel degradation, and porosity, are particularly important because they can alter local mechanical constraints, cell–matrix adhesion, and mechanotransduction, thereby driving morphological changes such as lumen formation and budding [173,174]. If these environmental variables are not adequately considered, AI models may correctly detect morphological alterations yet incorrectly attribute their biological origin. Thus, a key challenge for OIM is not merely to detect morphological changes, but to determine whether they primarily reflect intrinsic biological states or microenvironment-driven adaptations. This distinction is particularly difficult because microenvironmental cues are multidimensional, dynamic, and interdependent, making their individual contributions to organoid morphology difficult to isolate [175].

### 5.2 Algorithmic bottlenecks: computational cost, reproducibility, and interpretability

Even with high-quality datasets, current OIM algorithms face substantial computational and conceptual limitations that restrict their broader adoption. These challenges primarily involve computational scalability, model generalizability, reproducibility, and the ability to extract biologically meaningful insights beyond statistical prediction.

First, the computational demands associated with high-dimensional organoid imaging remain a major barrier. 3D organoid imaging generates large volumetric datasets that require substantial storage capacity, processing power, and specialized computational infrastructure. Advanced DL architectures often require extensive training data and considerable computational resources, limiting accessibility for laboratories without dedicated AI infrastructure. Although transfer learning and lightweight architectures provide potential solutions, achieving efficient analysis of complex organoid morphology while maintaining accuracy remains challenging.

A second challenge concerns algorithmic generalizability and reproducibility. Although many OIM models demonstrate impressive performance on internally generated datasets, their robustness across independent datasets, laboratories, and experimental conditions remains insufficiently validated (Table S2). Distribution shifts caused by differences in imaging systems, organoid types, and experimental protocols can substantially reduce model performance. Furthermore, variations in software environments, model initialization, optimization strategies, and preprocessing pipelines may hinder computational reproducibility, making it difficult to independently verify reported findings.

Finally, limited interpretability and the difficulty of deriving causal insights from DL models represent major challenges for biological discovery. The lack of model transparency limits researchers’ ability to extract mechanistic insights or formulate experimentally testable biological hypotheses from model outputs, potentially reducing sophisticated algorithms to statistical predictors without mechanistic understanding. Furthermore, current DL-based approaches predominantly capture statistical associations between morphological features and biological outcomes rather than distinguishing causal determinants from correlated features, thereby limiting the mechanistic depth of insights that OIM can currently provide.

### 5.3 Translational barriers: regulatory, data governance and ethical hurdles

The clinical and industrial translation of OIM requires addressing challenges beyond technological performance, particularly regarding regulatory validation, responsible data management, and ethical governance.

A obstacle is the absence of clearly defined regulatory frameworks for OIM-based platforms. Although regulatory agencies, including the U.S. Food and Drug Administration (FDA) and the European Medicines Agency (EMA), have developed general guidelines for AI-based medical technologies and alternative testing approaches, specific requirements for integrated organoid imaging and AI analysis pipelines remain unclear [176,177]. Depending on their intended applications, OIM systems may be classified differently, ranging from research tools supporting drug development to software-based diagnostic platforms. This regulatory uncertainty complicates the establishment of validation standards, performance benchmarks, and approval pathways.

Beyond regulatory considerations, data governance and ethical challenges represent critical barriers for clinical translation. PDOs provide unique opportunities for personalized medicine by linking morphological phenotypes with clinical information [45,178]; however, integrating high-dimensional imaging data with genomic, transcriptomic, and clinical datasets raises substantial privacy concerns. Although organoid-derived imaging features are not conventional identifiers, their combination with patient-specific metadata may increase the risk of re-identification [179]. Furthermore, the use of patient-derived biospecimens for training commercial AI systems raises unresolved questions regarding informed consent, data ownership, and benefit sharing [180,181]. Importantly, insufficient diversity in organoid biobanks may also introduce systematic biases, potentially limiting the equitable application of OIM-based personalized medicine across diverse patient populations [182].

Together, these challenges highlight that the future advancement of OIM requires not only improved algorithms but also coordinated efforts in biological standardization, data infrastructure, computational transparency, and responsible governance.

## 6 Future perspectives

**Fig. 13:**
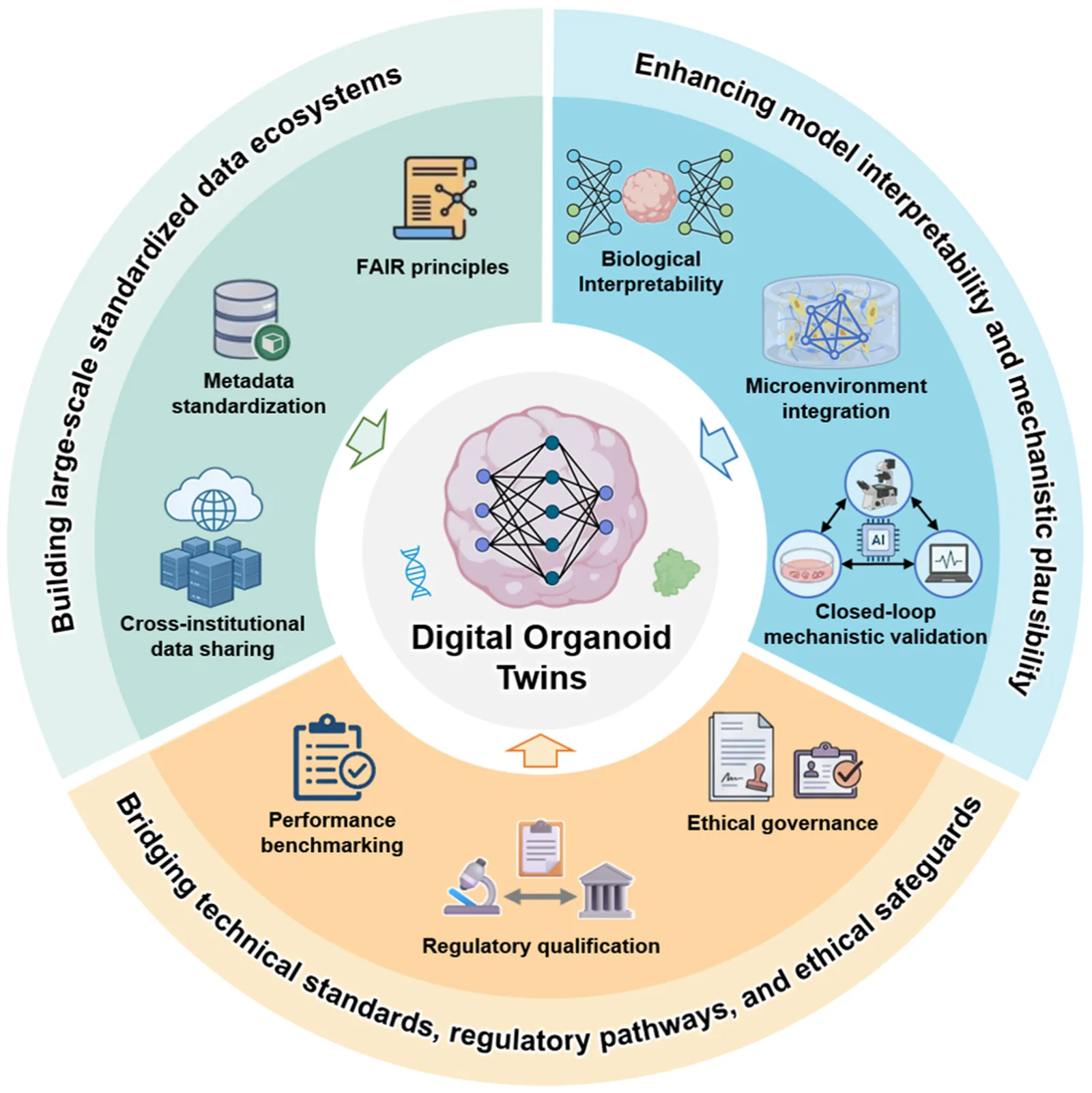
Overview of future perspectives for OIM. Future development of OIM may proceed along four interconnected directions: (1) Building large-scale standardized data ecosystems; (2) enhancing model interpretability and mechanistic plausibility; (3) establishing technical standards, regulatory pathways, data-governance frameworks, and ethical safeguards; and (4) integrating the organoid morphome with molecular and biomechanical information and physics-based simulations toward predictive digital organoid twins. Ultimately, physics-informed multimodal integration is poised to propel organoid research toward digital organoid twins.

Addressing these challenges will require future OIM development to move toward greater standardization, mechanistic interpretability, and translational robustness. Accordingly, four interconnected priorities can be identified: establishing large-scale and interoperable data infrastructures, improving model interpretability and mechanistic grounding, developing appropriate translational and governance frameworks, and integrating multimodal data with physics-based modeling to support predictive digital organoid twins (Fig. 13).

### 6.1 Building large-scale standardized data ecosystems

The long-term advancement of OIM will require a transition from fragmented, study-specific imaging datasets toward large-scale, standardized data ecosystems capable of supporting robust computational modeling. Although current OIM studies have demonstrated the feasibility of extracting quantitative morphological information from organoid images, the broader utility and generalizability of these approaches will increasingly depend on the availability of interoperable datasets that can be systematically shared, integrated, and reused across research communities.

A key priority in this transition is the establishment of data infrastructures guided by FAIR principles (findable, accessible, interoperable, and reusable) [183] that enable organoid imaging resources to evolve from isolated experimental outputs into shared, reusable computational resources. Beyond image storage, such infrastructures should incorporate standardized metadata frameworks capturing key experimental contexts, including organoid source, culture conditions, imaging parameters, and analytical workflows [184,185]. The development of shared ontologies and standardized reporting frameworks will be equally important for harmonizing terminology and experimental descriptions, thereby enabling datasets generated across different laboratories and platforms to be consistently annotated, meaningfully compared, and integrated [186,187]. However, technical standardization alone will not ensure sustained cross-institutional data sharing. Long-term data ecosystems will also require accessible repositories, standardized acquisition protocols, structured metadata capture, and clearly defined data-sharing practices that facilitate the deposition, curation, and reuse of datasets across research communities [188,189]. Such coordinated infrastructure will be essential for establishing a sustainable, community-wide data resource for OIM development.

Beyond facilitating data sharing and reuse, standardized and interoperable data resources could provide an important foundation for large-scale computational modeling of organoid morphology. Recently, foundation-model-based approaches are beginning to extend organoid image analysis beyond conventional task-specific paradigms. Whereas task-specific models are typically optimized for a predefined dataset and analytical objective [90,190], foundation models leverage broadly pretrained representations that can be adapted to diverse downstream tasks, potentially reducing the need for task-specific annotation and model development from scratch [191]. OrgLine provides an early example of this emerging paradigm in organoid analysis by integrating pretrained detection with prompt-guided segmentation within a unified morphometric workflow [192]. Although the transferability of such approaches remains dependent on diverse, well-curated datasets and robust adaptation across organoid systems and imaging conditions, foundation models could reduce reliance on separately trained models for individual applications and provide a more unified computational backbone for OIM [193,194]. It's worth to notification that the foundation models should be viewed as enabling computational technologies within OIM rather than as substitutes for the OIM framework itself. Their primary contribution is to strengthen the representation and analysis layer, while the broader OIM workflow remains centered on linking morphological representations to biological states and experimentally supported interpretation. Therefore, combined with standardized data infrastructures, this paradigm may ultimately facilitate the development of more scalable, transferable, and generalizable representations of the organoid morphome across organoid types, experimental conditions, and imaging platforms.

### 6.2 Enhancing model interpretability and mechanistic plausibility

Future models developed within the OIM framework will need to move beyond predictive performance alone toward computational systems that are both biologically interpretable and mechanistically plausible. Although current DL approaches can identify morphological signatures associated with developmental states, disease-associated changes, and therapeutic responses, high predictive accuracy does not necessarily imply that the learned features reflect the underlying biological drivers [195]. This limitation becomes particularly important because similar morphological alterations may arise from distinct intrinsic or microenvironmental mechanisms. Future OIM pipelines should therefore not only identify which morphological features are predictive, but also place these features in biological context by investigating why they arise and whether they correspond to biologically meaningful mechanisms.

One important direction is the development of interpretable representations that bridge computational features and biological processes. Rather than relying exclusively on high-dimensional latent representations that are difficult to interpret, future models could incorporate biologically informed feature learning to identify morphological patterns associated with specific cellular behaviors, tissue organization, or developmental trajectories. Integrating explainable AI (XAI) with quantitative morphomics may further reveal which structural features contribute most strongly to model predictions and whether these features correspond to experimentally validated biological mechanisms [196]. Biologically informed neural networks and related knowledge-guided learning strategies could complement such post hoc interpretation by incorporating prior biological constraints, developmental principles, or known regulatory relationships directly into model construction [[197], [198], [199]]. Collectively, these approaches could help shift OIM from the identification of statistical morphology–outcome associations toward the generation of biologically testable hypotheses.

A further opportunity is to incorporate mechanobiological and biomaterial descriptors into OIM. Quantitative variables such as matrix stiffness, viscoelasticity, degradability, porosity, ECM composition, hydrogel formulation, fluid shear stress, mechanical strain, and compressive stress could be integrated alongside image-derived morphological representations as contextual variables [172,184,200]. Sample-level factors such as matrix stiffness or hydrogel composition could be encoded as covariates [201], whereas spatially heterogeneous mechanical cues, such as stiffness gradients could be represented as spatially registered maps and integrated with image-derived morphological representations [202]. Such context-aware modeling could help account for microenvironment-associated morphological variation and clarify how morphological patterns relate to biological states within different physical environments. This integration would extend OIM from morphology-only inference toward morphomic interpretation within its material and biomechanical context.

Mechanistic interpretation will ultimately require experimental perturbation and validation rather than computational attribution alone. Controlled perturbation systems, including engineered matrices and organ-on-a-chip platforms, provide opportunities to systematically manipulate selected microenvironmental parameters, such as matrix stiffness, ECM composition, fluid shear stress, and cyclic strain, while monitoring the resulting morphological and functional responses [[203], [204], [205], [206]]. These controlled experiments could be incorporated into iterative model–experiment feedback loops in which computational models generate experimentally testable mechanistic hypotheses and associated predictions, targeted perturbations evaluate those predictions, and newly acquired imaging and biological measurements are used to refine the models [207,208]. By combining model-generated hypotheses with controlled perturbation experiments, this iterative strategy could test whether predicted morphology–biology relationships are experimentally reproducible under defined microenvironmental conditions. Such validation could help move OIM beyond predictive associations toward experimentally supported mechanistic interpretations of how microenvironmental factors shape organoid morphology and function.

### 6.3 Bridging technical standards, regulatory pathways, and ethical safeguards for OIM translation

The successful translation of OIM from proof-of-concept research into practical biomedical applications will require a transition from demonstrating technical feasibility toward establishing trustworthy and standardized computational systems. Although advances in DL and organoid technologies have accelerated the development of morphology-based prediction models, their adoption in research and clinical settings will ultimately depend on transparent evaluation frameworks, harmonized standards, and responsible governance structures.

A fundamental step toward this goal is the establishment of standardized criteria for evaluating analytical workflows developed within the OIM framework. Whereas conventional image-analysis evaluation often focuses on the performance of individual computational tasks, OIM requires assessment across an integrated analytical workflow spanning biological systems, imaging technologies, computational models, and experimental validation. Therefore, performance measured solely on internal validation datasets is insufficient for determining real-world reliability. Future efforts should promote multi-center benchmarking datasets, standardized reporting frameworks, and independent validation strategies that evaluate not only predictive accuracy but also robustness, reproducibility, and biological relevance. Such standards will provide a common foundation for comparing computational approaches and accelerating the transition from proof-of-concept studies toward reliable analytical platforms.

Regulatory evaluation will also need to accommodate the increasing integration of AI-driven analytics with organoid technologies. Regulatory assessment of OIM-enabled applications should extend beyond the performance of individual computational models to consider their intended use, risk profile, data provenance, end-to-end analytical workflow, and biological validation requirements. For example, OIM applications supporting drug discovery and preclinical evaluation may require different validation standards from those intended to inform clinical decision-making [209]. For clinically deployed applications, regulatory strategies may also need to address model updating, version control, and post-deployment performance monitoring to ensure continued reliability across changing data and experimental environments [210,211]. Establishing clear and adaptive regulatory pathways that balance innovation with reliability will therefore be essential for the responsible translation of OIM.

Equally important is the development of ethical safeguards for the responsible implementation of OIM. As OIM increasingly integrates PDOs with multidimensional imaging features, molecular profiles, and clinical information, robust data governance will become essential for maintaining privacy, transparency, and public trust [212,213]. Privacy-preserving computational strategies, transparent data-use policies, and equitable representation within organoid resources will be necessary to minimize potential biases and ensure that OIM-enabled precision medicine approaches benefit diverse patient populations. Importantly, ethical considerations should not be regarded as downstream constraints, but rather as foundational principles embedded throughout the design, validation, and deployment of OIM-enabled applications.

Together, these efforts will establish the foundation for trustworthy OIM translation. The broader impact of OIM will depend not only on the ability of AI models to accurately interpret organoid morphology, but also on whether OIM can meet the standards of reliability, transparency, clinical validity and responsible governance required for widespread adoption in biomedical research and clinical practice.

### 6.4 Towards digital organoid twins: physics-informed multimodal integration

A longer-term direction for OIM is to contribute to the development of artificial intelligence virtual organoids (AIVOs), in which organoid-scale biological states are represented and modeled *in silico* [214]. In this context, OIM could provide a morphology-centered state representation by converting longitudinal imaging data into quantitative descriptions of structural organization and temporal dynamics. However, morphology alone is insufficient to construct a comprehensive digital counterpart of an organoid. Future AIVOs will require integration of the organoid morphome with complementary molecular information, including transcriptomic, proteomic, and metabolomic profiles [215], as well as biomechanical and microenvironmental variables that influence organoid function [200].

The integration of AI with mechanistic computational models provides a potential route toward this goal. Rather than replacing mechanistic understanding with purely data-driven prediction, OIM could leverage AI to extract quantitative morphological states, integrate multimodal observations, and infer biologically relevant variables, while mechanistic models constrain how these states evolve under defined biological and physical conditions. Multi-scale computational frameworks, including vertex models [216], cellular Potts models [217], and PDE-based continuum models [218], provide potential foundations for representing cellular mechanics, multicellular organization, and tissue-scale dynamics, respectively. These approaches have increasingly been applied to investigate tissue and organoid morphogenesis, highlighting the potential of mechanistic modeling to connect morphological changes with underlying biological processes [219,220]. Within OIM framework, AI-driven morphomic analysis and mechanistic models could be synergistically combined to establish biologically constrained state-transition models, thereby bridging morphological pattern recognition and mechanistically grounded simulation.

Realizing a digital organoid twin will require an additional transition from static or one-time computational models toward dynamically updated counterparts of living organoids. A phased development pathway may therefore proceed from standardized longitudinal repositories that pair 4D imaging with molecular, biomechanical, and microenvironmental metadata, to hybrid AI–mechanistic models capable of forecasting short-term developmental or treatment-induced state transitions. As new experimental observations become available, these models could be recalibrated and updated to maintain correspondence with the evolving biological system. Such workflows will also require rigorous validation of model predictions, parameter calibration, and uncertainty assessment before being used for prospective perturbation analysis. Importantly, prediction of future states should be distinguished from *in silico* perturbation testing: the latter requires models that are sufficiently mechanistically constrained and experimentally validated to support counterfactual inference rather than merely reproduce statistical associations.

Within this progression, AIVOs may represent computational models of organoid states and dynamics, whereas a fully developed digital organoid twin would additionally require persistent correspondence with a defined biological counterpart through longitudinal observation, model updating, and experimental validation. Such a framework could ultimately enable *in silico* exploration of pharmacological, genetic, or physical perturbations before targeted wet-lab testing. In this sense, OIM would not itself constitute the digital twin, but would provide a morphology-centered analytical foundation for constructing and updating one. Ultimately, OIM could evolve from describing organoid morphology to representing dynamic biological states and, eventually, forecasting responses to defined perturbations through mechanistically constrained and experimentally validated computational models.

## 7 Conclusion

In this review, we introduce OIM as an integrative conceptual and analytical framework that transforms organoid morphology from qualitative observation into quantitative and predictive information. By organizing morphological quantification, functional association or prediction, and experimentally supported mechanistic validation into a hierarchical workflow, OIM highlights three major transitions in organoid analysis: from qualitative to quantitative, from static to dynamic, and from superficial observation to in-depth biological interpretation. Its applications in quality control, disease deconstruction, and drug development demonstrate the value of systematically integrating imaging and AI. This framework provides a structured basis for future methodological development and may facilitate more reproducible, scalable, and translational organoid research.

## CRediT authorship contribution statement

**Shangyan Li:** Writing – review & editing, Writing – original draft, Visualization. **Jiqing Huang:** Writing – review & editing, Writing – original draft, Visualization. **Yongyong Yan:** Writing – review & editing, Writing – original draft. **Richard T. Jaspers:** Writing – review & editing, Conceptualization. **Janak Lal Pathak:** Writing – review & editing, Funding acquisition. **Yin Xiao:** Writing – review & editing, Supervision, Conceptualization. **Qing Zhang:** Writing – review & editing, Supervision, Funding acquisition, Conceptualization.

## Ethics approval and consent to participate

Not applicable for this manuscript. This study did not involve animal/human participants, animal/human data, or animal/human tissue.

## Funding

This work was supported by the National Natural Science Foundation of China (32301129) and the Science and Technology Planning Project of Guangzhou, China (202201020203).

## Declaration of competing interest

The authors declare that they have no known competing financial interests or personal relationships that could have appeared to influence the work reported in this paper.
