# Supplementary material for: Organoid Intelligent Morphomics: Decoding the organoid morphome through artificial intelligence from phenotypic quantification to mechanistic insight
Source: Bioact Mater. 2026 Sep 3;68:162–87. doi: 10.1016/j.bioactmat.2026.08.043 (PMC13571529; doi:10.1016/j.bioactmat.2026.08.043)
Supplement: Supplementary file 1 [file mmc1.docx]

**Table S1. Conceptual comparison between OIM and related paradigms.** This table compares OIM with related biological, computational, and imaging paradigms in terms of their ontological nature, target objects, core objectives, distinctive features and limitations.

| **Paradigm** | **Ontological nature** | **Target object** | **Core objective** | **Distinctive features and limitations** | **References** |
| --- | --- | --- | --- | --- | --- |
| OIM | Integrative analytical framework | Live organoids | Decoding the morphome to establish predictive morphology-function-mechanism mapping | Integrates standardized morphological data acquisition, AI-driven multidimensional morphome construction, morphology–biology association and predictive modelling, and biological validation. | – |
| Phenomics | Holistic biological profiling framework | Biological systems across all scales | Systematic profiling of full-scale phenotypic traits under genetic or environmental perturbations | Broad phenotypic profiling; not specific to organoid morphology. | ^1^ ^2^ |
| Morphomics | Structural phenotypic analytical framework | Biological systems across all scales | Systematic study of morphological features within a biological system | Primarily characterizes spatial structural properties; traditionally emphasizes static shape representations. | ^3^ |
| Radiomics | Diagnostic analytical paradigms | Standard-of-care medical images | Non-invasive clinical disease diagnosis, subtyping, and treatment response prediction | Converts medical images into quantitative imaging phenotypes; largely association-based, with limited mechanistic insight. | ^4,5^ |
| Computational pathology | Diagnostic analytical paradigms | Histological sections | Diagnostic assistance and prognosis prediction | Operates on fixed, non-viable tissues; inherently static, precluding longitudinal tracking of live morphodynamics. | ^6,7^ |
| Organoid morphometrics | Quantitative geometric measurement methodology | Cellular/organoid structures | Quantification of basic geometric and morphological parameters | Enables objective quantification of size, shape, and other geometric properties; primarily descriptive and parameter-focused. | ^8^ |
| HCI | Automated imaging and quantification platforms | cells/organoids | Automated, high-throughput acquisition of multi-parametric image data | Excellent for data generation and throughput; primarily an acquisition technology rather than a analytical framework. | ^9,10^ |
| HCS | Automated screening platforms | cells/organoids | High-throughput assessment of drug efficacy or toxicity | Predominantly endpoint analysis; limited depth in structural/mechanistic insights. | ^11^ |
| Traditional organoid imaging analysis | Descriptive image processing methods | Raw microscopy images | Basic morphological assessment and qualitative endpoint evaluation | Subjective, labor-intensive, and low throughput; difficult to capture high-dimensional latent biological patterns or long-term morphodynamics. | ^8,12^ |
| AI-based organoid image analysis | Automated computer vision pipelines | Raw microscopy images | Automated analysis of organoid image | Automates detection, segmentation, classification, and quantitative image analysis; an analytical approach rather than an integrative framework. | ^13,14^ |
| AI-based phenotypic profiling/AI-assisted phenotyping | High-dimensional feature classification frameworks | cells/organoids | Identifying phenotypic signatures for screening | Broad but often phenomenological; does not necessarily require mapping back to specific molecular mechanisms or functionality. | ^15,16^ |
| Digital organoids | 3D virtual reconstructions | organoids | Digital characterization and analysis of organoids | Constructing virtual representations of organoid structure for visualization and computational analysis. | ^17^ |
| Virtual organoids | Generative synthetic image distributions | organoids | *In silico* reconstruction and simulation of organoid dynamics | Generates synthetic organoid images or representations for data augmentation, simulation, or virtual experimentation. | ^18,19^ |
| AIVOs | In silico multiscale biophysical simulation systems | organoids | AI-enabled digital twins for organoid prediction and virtual experimentation | Enables *in silico* representation and simulation of organoid states and responses by integrating AI with multiscale biological or biophysical modeling. | ^19^ |

***Abbreviations:*** *OIM, organoid intelligent morphomics; AI, artificial intelligence; AIVOs, artificial intelligence virtual organoids; HCI, high-content imaging; HCS, high-content screening; 4D, four-dimensional.*

**Table S2.** **Comparison of AI tools and applications in OIM.** This table provides a structured evaluation of representative AI tools and studies across key operational and performance dimensions, including AI task, organoid model, sample size, quantitative performance metrics, external validation, cross-center generalizability, code/data availability, and key limitations. It enables researchers to assess the computational robustness, translational readiness, and practical utility of each method, thereby supporting informed tool selection and highlighting critical methodological gaps in current OIM research.

| **Tool/study** | **AI task and Performance** | **Organoid model** | **Sample size** | **External validation** | **Cross-center tested** | **Code/data availability** | **Key limitations** | **References** |
| --- | --- | --- | --- | --- | --- | --- | --- | --- |
| Deliod | Detection and classification: mAP50 = 87.5% (on Dataset 1), mAP50 = 92.1% (on Dataset 2) | Intestinal organoid | 840 images (Dataset 1); 1,750 images, (Dataset 2) | Yes | No | No | Small dataset with class imbalance | ^20^ |
| 3D-Organoid-SwinNet | Segmentation: Dice = 94.91 ± 0.50, HD95 = 04.03 ± 0.05 | Breast cancer organoid | Not specified | No | No | Yes (GitHub) | Transformer models require large datasets | ^21^ |
| OrganoSeg2 | Segmentation: IoU = 0.906 (median); Tracking: Accuracy: 80–100% | Colon organoid, PDAC organoid | Total manual segments: N = 1051 | Yes | No | Yes (GitHub) | Challenges in tracking small and dense organoids | ^22^ |
| MOrgAna | Segmentation: Not specified | BO, intestinal organoid | 91 images | No | No | Yes (GitHub) | Relies on manually annotated training sets | ^23^ |
| CartoCell | Segmentation: (1) geometric features: Mean relative error = 5.6%; (2) connectivity features: Mean relative error = 13.3% | MDCK 3D epithelial cysts | Not specified | Yes | No | Yes (GitHub) | Limited adaptability to complex tissues | ^24^ |
| Organalysis | Feature Importance: Not specified; Success rate = 92% (noisy images) | Cardiac organoid | 46 images | No | No | No | Limitations in default format handling | ^25^ |
| ReViMS version 2.0 | Segmentation and volume estimation: AD% = 0.8% | Human lung adenocarcinoma spheroid | Not specified | No | No | Yes (SourceForge) | Subjectivity of manual expert segmentation | ^26^ |
| IEP-CDS | Detection and classification: F1-score = 0.488; mAP = 0.488 | Inner ear organoid | Original: 6 images | No | No | Yes (GitHub) | Extremely small sample size | ^27^ |
| TransOrga-plus | Segmentation: Dice = 0.919, mIoU = 0.851; Tracking: Accuracy = 92.1%–94.4%; Classification: Accuracy = 92.86% ± 2.28% | Salivary gland ACC organoid | 1,153 images | Yes | Yes | Yes (GitHub) | Insufficient detection accuracy for small targets | ^28^ |
| CytoCensus | Detection: F1-score = 0.96, F1-score = 0.98; Tracking: Not specified | Retinal organoid | Detection: 25images;  Tracking: Not specified | Yes | Yes | Yes (GitHub) | Requires initial user training | ^29^ |
| OrganoidTracker 2.0 | Detection: Accuracy: ~99% | Intestinal organoid | Not specified | Yes | No | Yes (GitHub) | High computational resource demand | ^30^ |
| Organoid App | Image prediction:SSIM up to ~15% | Artificial skin | 75 images | No | No | No | Extremely small dataset | ^31^ |
| AngioMT + REAVER | Regression (Predicting NVP & NOD): NVP: R² = 0.64, MAE = 0.14; NOD: R² = 0.74, MAE = 0.09 | Vascularized microphysiological system | Not specified | No | No | No | Single data source | ^32^ |
| VC-Net | Semantic segmentation: Dice = 0.81, IoU = 0.70, HD95 = 86.33 | Microvascular network | 1984 images | No | No | No | Limited dataset size | ^33^ |
| YOLO | Segmentation and Classification: mAP50 = 0.845; Precision = 0.787; Recall = 0.763 | Intestinal organoid | 840 images | No | No | No | Low segmentation accuracy in dense cultures with overlapping organoids | ^34^ |
| U-Net framework | Feature Correlation: Pearson r: 0.89–0.92; | Gastric cancer organoid | 756 image pairs | No | No | No | Model based on specific cell lines | ^35^ |
| FEMSSorg | Detection: AP₅₀ = 0.913 (Intestine), 0.916 (Lung), 0.989 (Brain); Classification: AP₅₀ = 0.783 (Intestine), 0.746 (Lung), 0.777 (Stomach) | Intestine, Lung organoid | Thousands of images, >120,000 annotated instances. | No | No | No | Boundary ambiguity problem | ^36^ |
| Cellos | Segmentation: Precision: 96.07%, Recall: 83.80%, F1: 89.52 | TNBC PDO | Not specified | Yes | No | Yes (GitHub) | Limited to fluorescent nuclear labeling | ^37^ |
| deepOrganoid | Regression (Actual vs. Predicted): Pearson r= 0.95–0.97 | CRC organoid | 3,456 images | Yes | No | Yes (GitHub) | High data demand | ^38^ |
| SAAVY | Segmentation: IoU = 0.622 (ECHO) / 0.653 (Operetta) | PDAC spheroids | Training: 24 images; validation: 416–2,376 images | Yes | No | Yes (GitHub) | Trained only on PDAC spheroids; relatively small training dataset | ^39^ |
| EGO-Net | Segmentation: accuracy = 85.4% | CRC organoid | Not specified | No | No | No | Limited sample size | ^40^ |
| 2D-SIGMAT | Classification: Recovery Efficiency =100% | CRC organoid | 39,516 images | No | No | No | Over-aggregation issues | ^41^ |
| DOSS | Classification: Overall Precision: > 90% | CRC organoid | Not specified | No | No | No | Single organoid source | ^42^ |
| 3D U-Net +SegFormer | Segmentation: 3D U-Net: Dice = 0.92 ± 0.06;  SegFormer: Dice = 0.91 ± 0.11 | BO | Not specified | No | No | Yes (GitHub) | Relatively small dataset | ^43^ |
| Modified 3 model | Classification: Accuracy = 0.90 (EfficientNet-B0), 0.99 (B1), 1.00 (B2); F1-score = 1.00 | Breast cancer organoid | 2,383 images | No | No | No | Generalization not fully validated | ^44^ |
| Trainable Weka Segmentation | Segmentation: Mean Absolute Error = 0.0181, Root Mean Squared Error = 0.0827, Relative Absolute Error = 4.32% | Mouse heart organoid | Not specified | No | No | No | Specific to heart organoids | ^45^ |
| D-CryptO | Classification: Opacity: Accuracy = 98.00%; Budding: Accuracy = 90.87% | Colon organoid | Opacity: 2,478 images; Budding: 2,476 images | No | No | Yes (OSF) | Specifically tailored to colon organoids | ^46^ |
| OrgaSegment | Segmentation: AP= 0.76 ± 0.12 (at IoU = 0.5) | PDIO | 231 images | Yes | Yes | Yes (GitHub) | Difficulty segmenting highly irregular organoids | ^47^ |
| EfficientNetV2-S+ Vision Transformer | Classification：EfficientNetV2-S: Accuracy = 67.3%; Vision Transformer：Accuracy = 65.7% | Hypothalamic-pituitary organoid | 1,500 images | Yes | No | Yes (GitHub) | Relatively limited sample size | ^48^ |
| VGG19-basedmodel | Classification: Accuracy = 79%, AUROC = 0.83 | Hypothalamic-pituitary organoid | 935–1,407 images per time point | Yes | No | Yes (Zenodo) | Derived from a single reporter cell line | ^49^ |
| ResNet50v2 | Classification: AUROC = 0.91, Accuracy = 84%, F1 = 0.89, Sensitivity = 0.85, Specificity = 0.82 | Retinal organoid | 1,209 images | No | No | No | Limited to binary classification tasks | ^50^ |
| Ensemble of CNNs | Segmentation: Not specified  Classification: F1-score > 0.70 | Retinal organoid | Segmentation: 841 images; Classification:117,249 images total | Yes | No | Yes (GitHub) | Specific to retinal organoids | ^51^ |
| ResNet50 / DenseNet121, etc. | Regression prediction: RMSE = 1.014, MAE = 0.740, MAPE = 4.010%, Correlation = 0.808 | Airway organoid | Not specified | No | No | No | No external validation; small sample size | ^52^ |
| SCOUT | Segmentation: Accuracy = 90%; Classification: Dice = 97.2% | Human cerebral organoid | Segmentation: Not specified; Classification:7,596 images | No | No | Yes (GitHub) | Long data processing time; limited number of antibody markers | ^53^ |
| Sequential deep learning model) | Classification: Not specified; Regression prediction: pearson r= 0.78 | PDTO | 20 images per label | No | No | Yes (GitHub) | Limited sample size | ^54^ |
| AI-based classifier | Classification: Not specified | CRC PDO | 984 images | No | No | No | Classification accuracy can be further improved | ^55^ |
| Segment Anything Model (v1.0) | Segmentation: Not specified;  Classification: Accuracy = 82% | OSCC organoid | 68 images | Yes | No | Yes (GitHub) | Molecular mechanisms to be elucidated | ^56^ |
| POST | Segmentation: AP = 0.81–0.89 | Pancreatic cancer, lung cancer organoid | 1,362 images | No | No | Yes (GitHub) | Brightfield imaging only | ^57^ |
| CNN | Classification: Z' factor = ~1 | Neural organoid | Training: 3,415 images; Validation: 1,464 images | Yes | No | Yes (GitHub) | Lacks animal model validation | ^58^ |
| DILITracer | Classification: Accuracy = 82.34% | Human liver organoid | 12-20 z-axis images per day | No | No | Yes (GitHub) | Class imbalance in samples; single donor source | ^59^ |
| U-Net+ AlexNet | Segmentation: Not specified; Classification: Accuracy >95% | Kidney organoid | 1,758 images | No | No | No | Limited training data; handling of adherent organoids | ^60^ |
| Random forest classifier | Classification: Accuracy = 86% | Human midbrain organoid | Not specified | No | No | Yes (GitHub) | Relatively limited sample size; only addresses a single toxicity mechanism | ^61^ |
| ImPheNet | Classification: Accuracy = 0.967, Precision = 0.95 | BO | 1,619 images | No | No | Yes (Zenodo) | Limited sample size; imaging depth limitations | ^62^ |
| Orbits | Segmentation: Not specified; Cell death detection: Z' factor = 0.71 | PDAC PDO | Not specified | No | No | No | Small sample size | ^63^ |
| U-Net+ TrackMate (v7.6.1) | Segmentation: IoU = 0.897 ± 0.109 (95% confidence level)  Classification: AUC = 0.966 ± 0.020 | Breast cancer organoid | Segmentation: 100 imaging frames; Classification training: Not specified | No | No | Yes (GitHub) | Impact of bioprinting on cells; limitations of imaging focal plane | ^64^ |
| OrgLine | Detection: AP₀.₅₋₀.₉₅ = +9.94% vs. Tellu; Segmentation: DSC > 0.91; Classification:  Accuracy 78.8% | Intestinal, brain, lung, colon, PDAC, stomach, breast organoid | ~8,000 images | No | Yes | Yes (GitHub) | Insufficient data diversity and scale; false positives/ negatives still exist | ^65^ |

***Abbreviations:*** *AI, artificial intelligence; CNN, convolutional neural network; DL, deep learning; ML, machine learning; DILI, drug-induced liver injury; 3D, three-dimensional; 3D-oids, 3D models including spheroids, organoids, tumoroids, and assembloids; PDO, patient-derived organoid; PDIO, patient-derived intestinal organoid; PDTO, patient-derived tumor organoid; ACC, adenoid cystic carcinoma; BO, brain organoid; CCO, colorectal cancer organoid; CRC, colorectal cancer; NSCLC, non-small cell lung cancer; OSCC, oral squamous cell carcinoma; PDAC, pancreatic ductal adenocarcinoma; TNBC, triple-negative breast cancer; MDCK, Madin–Darby canine kidney; BF, brightfield microscopy; OCT, optical coherence tomography; MRI, magnetic resonance imaging; OSF, open science framework; AP, average precision; mAP, mean Average Precision;* *AUC, area under the curve; IoU, intersection over union; TP, true positive; Z' factor, Z-prime factor;* *AD,* *percentage Absolute Difference; SSIM: Structural Similarity Index; NVP, Normalized Vascular Potential; NOD, Normalized Oxygen Delivery; MAPE, Mean Absolute Percentage Error; MAE, Mean Absolute Error; DSC , Dice Similarity Coefficient; HD95, Hausdorff Distance at 95%; mIoU, mean Intersection over Union; AP₅₀, Average Precision at IoU=0.5; RMSE, Root Mean Square Error.*

**References**

1	Henderson, D. *et al.* The morphome and EDI in anatomy education: A systematic scoping review and expert recommendations. *Annals of anatomy = Anatomischer Anzeiger : official organ of the Anatomische Gesellschaft* **266**, 152829 (2026). <https://doi.org/10.1016/j.aanat.2026.152829>

2	Liu, Y. *et al.* Mapping Cell Phenomics with Multiparametric Flow Cytometry Assays. *Phenomics (Cham, Switzerland)* **2**, 272-281 (2022). <https://doi.org/10.1007/s43657-021-00031-0>

3	Mayhew, T. M. & Lucocq, J. M. From gross anatomy to the nanomorphome: stereological tools provide a paradigm for advancing research in quantitative morphomics. *Journal of anatomy* **226**, 309-321 (2015). <https://doi.org/10.1111/joa.12287>

4	Netti, S. *et al.* Methodological issues in radiomics: impact on accuracy of MRI for predicting response to neoadjuvant chemotherapy in breast cancer. *European radiology* **35**, 4325-4334 (2025). <https://doi.org/10.1007/s00330-024-11260-y>

5	Lambin, P. *et al.* Radiomics: the bridge between medical imaging and personalized medicine. *Nature Reviews Clinical Oncology* **14**, 749-762 (2017). <https://doi.org/10.1038/nrclinonc.2017.141>

6	Tan-Garcia, A., Chua, T. H. & Leow, W. Q. Computational pathology in the age of artificial intelligence - embrace not fear. *The journal of pathology. Clinical research* **11**, e70049 (2025). <https://doi.org/10.1002/2056-4538.70049>

7	Hölscher, D. L. & Bülow, R. D. Decoding pathology: the role of computational pathology in research and diagnostics. *Pflugers Archiv : European journal of physiology* **477**, 555-570 (2025). <https://doi.org/10.1007/s00424-024-03002-2>

8	Zubova, A. V. *et al.* Advances in organoid imaging and automated morphometric analysis: from optical microscopy to computational approaches. *Front Cell Dev Biol* **14**, 1861934 (2026). <https://doi.org/10.3389/fcell.2026.1861934>

9	Comolet, G. *et al.* A highly efficient, scalable pipeline for fixed feature extraction from large-scale high-content imaging screens. *iScience* **27**, 111434 (2024). <https://doi.org/10.1016/j.isci.2024.111434>

10	Carreras-Puigvert, J. & Spjuth, O. Artificial intelligence for high content imaging in drug discovery. *Current opinion in structural biology* **87**, 102842 (2024). <https://doi.org/10.1016/j.sbi.2024.102842>

11	Massei, R. *et al.* High-content screening (HCS) workflows for FAIR image data management with OMERO. *Scientific reports* **15**, 16236 (2025). <https://doi.org/10.1038/s41598-025-00720-0>

12	Du, X. *et al.* Organoids revealed: morphological analysis of the profound next generation in-vitro model with artificial intelligence. *Bio-Design and Manufacturing* **6**, 319-339 (2023). <https://doi.org/10.1007/s42242-022-00226-y>

13	Park, T. *et al.* Development of a deep learning based image processing tool for enhanced organoid analysis. *Scientific reports* **13**, 19841 (2023). <https://doi.org/10.1038/s41598-023-46485-2>

14	Xu, Y. *et al.* The application and progress of AI-based image analysis in tumor organoid research. *iScience* **29** (2026). <https://doi.org/10.1016/j.isci.2026.116756>

15	Grys, B. T. *et al.* Machine learning and computer vision approaches for phenotypic profiling. *J Cell Biol* **216**, 65-71 (2017). <https://doi.org/10.1083/jcb.201610026>

16	Hark, R. *et al.* AI-assisted phenotyping in a zebrafish hypophosphatasia model enables early and precise detection of skeletal alterations. *Scientific reports* **15**, 32578 (2025). <https://doi.org/10.1038/s41598-025-19199-w>

17	Yang, Q. *et al.* A New Perspective on Precision Medicine: The Power of Digital Organoids. *Biomaterials research* **29**, 0171 (2025). <https://doi.org/10.34133/bmr.0171>

18	Song, E. *et al.* VONet: A deep learning network for 3D reconstruction of organoid structures with a minimal number of confocal images. *Patterns (N Y)* **5**, 101063 (2024). <https://doi.org/10.1016/j.patter.2024.101063>

19	Bai, L. & Su, J. Artificial Intelligence Virtual Organoids (AIVOs). *Bioactive materials* **59**, 45-68 (2026). <https://doi.org/10.1016/j.bioactmat.2025.12.030>

20	Sun, Y. *et al.* Deliod a lightweight detection model for intestinal organoids based on deep learning. *Scientific reports* **15** (2025). <https://doi.org/10.1038/s41598-025-89409-y>

21	Sohaib, M., Shabani, S., Mohammed, S. A. & Parvin, B. 3D-Organoid-SwinNet: High-Content Profiling of 3D Organoids. *IEEE JOURNAL OF BIOMEDICAL AND HEALTH INFORMATICS* **29**, 792-798 (2025). <https://doi.org/10.1109/JBHI.2024.3511422>

22	Wells, C. J., Labban, N., Showalter, S. L., Przanowska, R. K. & Janes, K. A. Fast learning-free organoid quantification and tracking with OrganoSeg2. *Scientific reports* **16** (2026). <https://doi.org/10.1038/s41598-026-37526-7>

23	Gritti, N. *et al.* MOrgAna: accessible quantitative analysis of organoids with machine learning. *DEVELOPMENT* **148** (2021). <https://doi.org/10.1242/dev.199611>

24	Andres-San Roman, J. A. *et al.* CartoCell, a high-content pipeline for 3D image analysis, unveils cell morphology patterns in epithelia. *Cell reports methods* **3**, 100597-100597 (2023). <https://doi.org/10.1016/j.crmeth.2023.100597>

25	Singaraju, J. P. *et al.* Organalysis: Multifunctional Image Preprocessing and Analysis Software for Cardiac Organoid Studies. *Tissue Eng. Part C-Methods* **29**, 572-582 (2023). <https://doi.org/10.1089/ten.tec.2023.0150>

26	De Santis, I., Tasnadi, E., Horvath, P., Bevilacqua, A. & Piccinini, F. Open-Source Tools for Volume Estimation of 3D Multicellular Aggregates. *Appl. Sci.-Basel* **9** (2019). <https://doi.org/10.3390/app9081616>

27	Li, Y.-T., Chen, C.-Y., Wang, B.-S., Hung, P.-H. & Lin, C.-Y. Inner Ear Multiple Primary Cell Type Detection System. *Sci. Data* **12** (2025). <https://doi.org/10.1038/s41597-025-04700-x>

28	Qin, Y. M. *et al.* A knowledge-driven deep learning framework for organoid morphological segmentation and characterization. *Bmc Biology* **23**, 14 (2025). <https://doi.org/10.1186/s12915-025-02411-8>

29	Hailstone, M. *et al.* CytoCensus, mapping cell identity and division in tissues and organs using machine learning. *eLife* **9** (2020). <https://doi.org/10.7554/eLife.51085>

30	Betjes, M. A., Kok, R. N. U., Tans, S. J. & van Zon, J. S. Cell tracking with accurate error prediction. *Nature methods* **22**, 2400-2410 (2025). <https://doi.org/10.1038/s41592-025-02845-6>

31	Stueve, P. *et al.* Analysis of organoid and immune cell co-cultures by machine learning-empowered image cytometry. *FRONTIERS IN MEDICINE* **10** (2024). <https://doi.org/10.3389/fmed.2023.1274482>

32	Tronolone, J. J., Mathur, T., Chaftari, C. P. & Jain, A. Evaluation of the morphological and biological functions of vascularized microphysiological systems with supervised machine learning. *bioRxiv : the preprint server for biology* (2023). <https://doi.org/10.1101/2023.01.12.523755>

33	Xu, L. *et al.* Label-free prediction of vascular connectivity in perfused microvascular networks in vitro. *Computer methods and programs in biomedicine* **271**, 108953 (2025). <https://doi.org/10.1016/j.cmpb.2025.108953>

34	Conte, L., De Nunzio, G., Raso, G. & Cascio, D. Multi-Class Segmentation and Classification of Intestinal Organoids: YOLO Stand-Alone vs. Hybrid Machine Learning Pipelines. *Applied Sciences-Basel* **15**, 16 (2025). <https://doi.org/10.3390/app152111311>

35	Yuan, Y. C. *et al.* Deep Learning-Powered Scalable Cancer Organ Chip for Cancer Precision Medicine. *Advanced science (Weinheim, Baden-Wurttemberg, Germany)*, e16660 (2026). <https://doi.org/10.1002/advs.202516660>

36	Deng, X. *et al.* Fuzzy Mixture-of-Experts Aggregation for Organoid Identification With Multiscale State Space Features. *IEEE Trans. Fuzzy Syst.* **34**, 324-335 (2026). <https://doi.org/10.1109/tfuzz.2025.3622935>

37	Mukashyaka, P. *et al.* Cellos: High-throughput deconvolution of 3D organoid dynamics at cellular resolution for cancer pharmacology. *bioRxiv : the preprint server for biology* (2023). <https://doi.org/10.1101/2023.03.03.531019>

38	Powell, R. T. *et al.* deepOrganoid: A brightfield cell viability model for screening matrix-embedded organoids. *SLAS DISCOVERY* **27**, 175-184 (2022). <https://doi.org/10.1016/j.slasd.2022.03.004>

39	Trettner, K. J., Hsieh, J., Xiao, W. K., Lee, J. S. H. & Armani, A. M. Nondestructive, quantitative viability analysis of 3D tissue cultures using machine learning image segmentation. *Apl Bioengineering* **8**, 11 (2024). <https://doi.org/10.1063/5.0189222>

40	Zhang, L. *et al.* Quantifying the drug response of patient-derived organoid clusters by aggregated morphological indicators with multi-parameters based on optical coherence tomography. *Biomed. Opt. Express* **14**, 1703-1717 (2023). <https://doi.org/10.1364/BOE.486666>

41	Paul, R. *et al.* Versatile Image-Assisted Cell Sorting by Selective Trapping with Spatiotemporal Multiparameter Targeting. *Acs Sensors* (2025). <https://doi.org/10.1021/acssensors.5c01433>

42	Zhao, Y. *et al.* DOSS: Microfluidic engineered system enables automated intelligent sorting and chemo-evaluation of droplet-based colorectal adenocarcinoma organoids. *Chem. Eng. J.* **522** (2025). <https://doi.org/10.1016/j.cej.2025.166597>

43	Deininger, L. *et al.* An AI-based segmentation and analysis pipeline for high-field MR monitoring of cerebral organoids. *Scientific Reports* **13**, 9 (2023). <https://doi.org/10.1038/s41598-023-48343-7>

44	Yun, E., Kim, J. & Jeong, D. Lightweight Deep Learning Model for Classification of Normal and Abnormal Vasculature in Organoid Images. *Sensors* **26**, 112 (2025). <https://doi.org/10.3390/s26010112>

45	Kaneko, R., Ishino, F. & Lee, J. Quantitative Imaging Analysis of Internal Structural Complexity in Mouse Heart Organoids; Comparison to Mouse Embryonic Hearts. *Genes Cells* **30**, 15 (2025). <https://doi.org/10.1111/gtc.70055>

46	Abdul, L. *et al.* D-CryptO: deep learning-based analysis of colon organoid morphology from brightfield images. *Lab on a chip* **22**, 4118-4128 (2022). <https://doi.org/10.1039/d2lc00596d>

47	Lefferts, J. W. *et al.* OrgaSegment: deep-learning based organoid segmentation to quantify CFTR dependent fluid secretion. *Communications Biology* **7** (2024). <https://doi.org/10.1038/s42003-024-05966-4>

48	Asano, T. *et al.* A deep learning approach to predict differentiation outcomes in hypothalamic-pituitary organoids. *Communications biology* **7** (2024). <https://doi.org/10.1038/s42003-024-07109-1>

49	Matsumoto, R., Suga, H., Takahashi, Y., Aoi, T. & Yamamoto, T. Prediction of the hypothalamus-pituitary organoid formation using machine learning. *Cell reports methods* **5**, 101119-101119 (2025). <https://doi.org/10.1016/j.crmeth.2025.101119>

50	Kegeles, E., Naumov, A., Karpulevich, E. A., Volchkov, P. & Baranov, P. Convolutional Neural Networks Can Predict Retinal Differentiation in Retinal Organoids. *FRONTIERS IN CELLULAR NEUROSCIENCE* **14** (2020). <https://doi.org/10.3389/fncel.2020.00171>

51	Afting, C. *et al.* A deep learning-based computational pipeline predicts developmental outcome in retinal organoids. *PLoS. Biol.* **24**, 32 (2026). <https://doi.org/10.1371/journal.pbio.3003597>

52	Lim, M. H. *et al.* Deep Learning Model for Predicting Airway Organoid Differentiation. *Tissue Eng. Regen. Med.* **20**, 1109-1117 (2023). <https://doi.org/10.1007/s13770-023-00563-8>

53	Albanese, A. *et al.* Multiscale 3D phenotyping of human cerebral organoids. *Scientific reports* **10** (2020). <https://doi.org/10.1038/s41598-020-78130-7>

54	Takagi, K., Takagi, M., Hiyama, G. & Goda, K. A deep-learning model for characterizing tumor heterogeneity using patient-derived organoids. *Scientific reports* **14** (2024). <https://doi.org/10.1038/s41598-024-73725-w>

55	Okamoto, T. *et al.* Integration of human inspection and artificial intelligence-based morphological typing of patient-derived organoids reveals interpatient heterogeneity of colorectal cancer. *CANCER SCIENCE* **113**, 2693-2703 (2022). <https://doi.org/10.1111/cas.15396>

56	Lee, M. R. *et al.* Organoid morphology-guided classification for oral cancer reveals prognosis. *CELL REPORTS MEDICINE* **6** (2025). <https://doi.org/10.1016/j.xcrm.2025.102129>

57	Du, X. *et al.* Precision organoid segmentation technique (POST): accurate organoid segmentation in challenging bright-field images. *Bio-Design and Manufacturing* **9**, 80-93 (2026). <https://doi.org/10.1631/bdm.2500119>

58	Metzger, J. J. *et al.* Deep-learning analysis of micropattern-based organoids enables high-throughput drug screening of Huntington's disease models. *Cell reports methods* **2**, 100297-100297 (2022). <https://doi.org/10.1016/j.crmeth.2022.100297>

59	Tan, S. *et al.* Development of an AI model for DILI-level prediction using liver organoid brightfield images. *Communications biology* **8** (2025). <https://doi.org/10.1038/s42003-025-08205-6>

60	Wang, Q. *et al.* High-throughput "read-on-ski" automated imaging and label-free detection system for toxicity screening of compounds using personalised human kidney organoids. *JOURNAL OF ZHEJIANG UNIVERSITY-SCIENCE B* **23**, 564-577 (2022). <https://doi.org/10.1631/jzus.B2100701>

61	Monzel, A. S. *et al.* Machine learning-assisted neurotoxicity prediction in human midbrain organoids. *Parkinsonism & related disorders* **75**, 105-109 (2020). <https://doi.org/10.1016/j.parkreldis.2020.05.011>

62	Turpin-Moreno, I. *et al.* Variability vs. phenotype: Multimodal analysis of Dravet syndrome brain organoids powered by deep learning. *Iscience* **28**, 22 (2025). <https://doi.org/10.1016/j.isci.2025.113831>

63	Le Compte, M. *et al.* Single-organoid analysis reveals clinically relevant treatment-resistant and invasive subclones in pancreatic cancer. *NPJ Precis Oncol* **7**, 128 (2023). <https://doi.org/10.1038/s41698-023-00480-y>

64	Tebon, P. J. *et al.* Drug screening at single-organoid resolution via bioprinting and interferometry. *Nature communications* **14** (2023). <https://doi.org/10.1038/s41467-023-38832-8>

65	Deng, X. *et al.* OrgLine: A versatile pipeline for organoid morphometry using detector-guided prompts. *Cell Rep Methods*, 101482 (2026). <https://doi.org/10.1016/j.crmeth.2026.101482>
